# Symbiosis preservation: Putative regulation of fatty acyl-CoA reductase by miR-31a within the symbiont harboring bacteriome through tsetse evolution

**DOI:** 10.3389/fmicb.2023.1151319

**Published:** 2023-04-11

**Authors:** Mason H. Lee, Gangqing Hu, Rita V. M. Rio

**Affiliations:** ^1^Department of Biology, Eberly College of Arts and Sciences, West Virginia University, Morgantown, WV, United States; ^2^Department of Microbiology, Immunology, and Cell Biology, West Virginia University School of Medicine, Morgantown, WV, United States

**Keywords:** tsetse, microRNA, evolution, fatty acyl-CoA reductase, symbiosis

## Abstract

Tsetse flies are the sole vectors of African trypanosomes. In addition to trypanosomes, tsetse harbor obligate *Wigglesworthia glossinidia* bacteria that are essential to tsetse biology. The absence of *Wigglesworthia* results in fly sterility, thus offering promise for population control strategies. Here, microRNA (miRNAs) and mRNA expression are characterized and compared between the exclusive *Wigglesworthia*-containing bacteriome and adjacent aposymbiotic tissue in females of two evolutionarily distant tsetse species (*Glossina brevipalpis* and *G. morsitans*). A total of 193 miRNAs were expressed in either species, with 188 of these expressed in both species, 166 of these were novel to Glossinidae, and 41 miRNAs exhibited comparable expression levels between species. Within bacteriomes, 83 homologous mRNAs demonstrated differential expression between *G. morsitans* aposymbiotic and bacteriome tissues, with 21 of these having conserved interspecific expression. A large proportion of these differentially expressed genes are involved in amino acid metabolism and transport, symbolizing the essential nutritional role of the symbiosis. Further bioinformatic analyses identified a sole conserved miRNA::mRNA interaction (miR-31a::fatty acyl-CoA reductase) within bacteriomes likely catalyzing the reduction of fatty acids to alcohols which comprise components of esters and lipids involved in structural maintenance. The *Glossina* fatty acyl-CoA reductase gene family is characterized here through phylogenetic analyses to further understand its evolutionary diversification and the functional roles of members. Further research to characterize the nature of the miR-31a::fatty acyl-CoA reductase interaction may find novel contributions to the symbiosis to be exploited for vector control.

## Introduction

Tsetse flies (family Glossinidae) are strict vertebrate blood feeders found in sub-Saharan Africa. Tsetse are the biological vectors of the medically and economically significant African trypanosomes (*Trypanosoma* spp.), the etiological agents of Human African Trypanosomiasis (HAT) and Animal African Trypanosomiasis (AAT), respectively, (Krafsur, 2009). Due to the high antigenic variation displayed by trypanosomes coupled with the lack of safe therapeutics to address infections within their mammalian hosts (Cross, 1978), the suppression of African trypanosomiasis relies heavily on tsetse population control.

All tsetse flies harbor a monoculture of an obligate mutualist, the Gammaproteobacterium *Wigglesworthia glossinidia* (Aksoy, 1995), within a bacteriome organ located at the anterior midgut. Here, *Wigglesworthia* is intracellular within specialized epithelial cells known as bacteriocytes. The ancient *Wigglesworthia*-tsetse symbiosis dates back 50–80 million years and likely enabled tsetse’s dietary ecology (Chen et al., 1999). *Wigglesworthia* is necessary to fulfill important biological roles for tsetse, including supplementing B-vitamins lacking in the blood-only diet and for immunological priming of larvae (Weiss et al., 2012; Michalkova et al., 2014; Snyder and Rio, 2015; Rio et al., 2019). Importantly, the absence of *Wigglesworthia* results in tsetse sterility (Nogge, 1976). Even as we expand our understanding of the functional significance of the critical tsetse-*Wigglesworthia* symbiosis, fundamental questions remain about how interspecies homeostasis is maintained throughout development and during energetically expensive life events such as pregnancy and trypanosome infection, as these events are associated with differences in metabolic demands. It is likely that homeostasis is coordinated by a multitude of mechanisms including epigenetic regulation mediated by microRNAs (miRNAs) (Feng et al., 2018, 2019; Sun et al., 2022).

MiRNAs are small (∼22 nt) non-coding RNAs involved in *post*-transcriptional regulation. Regulation occurs by miRNA binding to mRNAs through complementary base pair interactions, typically at the 3′ untranslated region (UTR) by the miRNA’s seed region (i.e., the 2nd–7th nucleotides at the miRNA 5′ end), enabling the formation of an RNA-induced silencing complex (RISC) and typically resulting in mRNA instability and degradation (Zhang et al., 2015). MiRNAs have been implicated in nearly all biological contexts with high conservation observed across eukaryotes (Song et al., 2004)which eases their identification into large families based on homology. MiRNAs have additionally emerged as potential regulators of insect-bacteria homeostasis (Zhang et al., 2015; Feng et al., 2018, 2019; Mukherjee et al., 2020; Sun et al., 2022).

In this study, we use high-throughput Illumina sequencing to identify and characterize tsetse miRNAs and mRNAs expression landscapes in the bacteriome compared to aposymbiotic tissue in two evolutionary distant species of tsetse flies (*Glossina brevipalpis* and *G. morsitans*) under the nutrient stress of pregnancy. Putative miRNA::mRNA interactions are predicted “*in silico”* and their significance toward fly biology and the tsetse-*Wigglesworthia* symbiosis discussed. We hypothesize that miRNA::mRNA interactions conserved between these distant tsetse species and differentially expressed in the bacteriome of pregnant flies relative to aposymbiotic and virgin tissue are candidates for regulatory mechanisms and biological pathways significant to the maintenance of *Wigglesworthia* during host reproduction. Here, we report on a putative fatty acyl-CoA reductase (FAR) gene (identified as GMOY009401 and GBRI041870 in *G. morsitans* and *G. brevipalpis*, respectively), likely regulated by miR-31a and playing a significant role in the symbiosis during tsetse fecundity. This finding prompted subsequent phylogenetic and structural analyses of the FAR gene family within Glossinidae to expand on its evolution and function. The discovery of these miRNAs::mRNAs-based regulatory pathways provide novel avenues for further functional analyses, and, ultimately targets for impeding tsetse fly fitness and trypanosome transmission.

## Materials and methods

### Insect rearing

*G. morsitans* and *G. brevipalpis* are maintained in an insectary at the West Virginia University Department of Biology on a 12-h day and light schedule. Flies are fed defibrinated bovine blood (Hemostat, Dixon, CA, USA) through an artificial membrane every 48 h (Moloo, 1971). Pupae were placed in individual containers to ensure age and mating status at time of RNA isolation.

### Dissection, purification of RNA, and library preparation

Bacteriome and aposymbiotic tissues (consisting of the crop and proventriculus immediately anterior to the bacteriome) were collected from age-matched females of known mating status and pooled to generate samples for RNA sequencing. To ensure that the miRNAs and mRNAs expressed throughout pregnancy were adequately profiled, an equivalent number of tissues from females containing 1st–3rd instar larva *in utero* were obtained. With *G. brevipalpis*, only tissues of mated females were collected, as pregnancy was the state of interest in this study coupled with the fastidious colony rearing of this species making sample acquisition more challenging in comparison to *G. morsitans*. The bacteriomes or aposymbiotic tissues of 20 tsetse females were pooled for one biological sample, resulting in a total of 9 biological samples within our analyses. Samples were dissected and stored in RNAlater Stabilization Solution (Invitrogen, Carlsbad, CA, USA) following the manufacturer’s instructions prior to RNA isolation. The bacteriomes and aposymbiotic tissue were homogenized using mechanical pestles, and total RNA was isolated using the PureLink RNA Mini Kit (Ambion, Austin, TX, USA) following the “Purifying RNA from Animal Tissues” protocol and subsequently treated with Turbo DNAse I (Invitrogen, Carlsbad, CA, USA). RNA quality was determined with an Agilent 2,000 Bioanalyzer RNA Nano chip before sequencing. mRNA libraries were built using KAPA-stranded mRNA library prep (Roche Diagnostic, Indianapolis, IN, USA) starting with 300–500 ng total RNA and 9 cycles of PCR. Pair-End Tags (PETs) with 50 bps on each end were sequenced on an Illumina HiSeq 2,500 (Marshall Genomics Core Facility). Small RNA libraries were built using NextFlex V3 Kit from Bioo Scientific (PerkinElmer Waltham, MA, USA) starting with 100 ng total RNA, 1/3rd adapter dilution, followed by 18 cycles of PCR. These samples were sequenced on an Illumina HiSeq 2,500 with single end 40 bp reads. Raw sequencing data were submitted to National Center for Biotechnology Information (NCBI) Sequence Read Archives (SRP408059).

### Data analysis

Adapter sequences were identified with FastQC and removed using TrimGalore v0.4.3.^1^

### mRNA analyses

**FIGURE 1:**
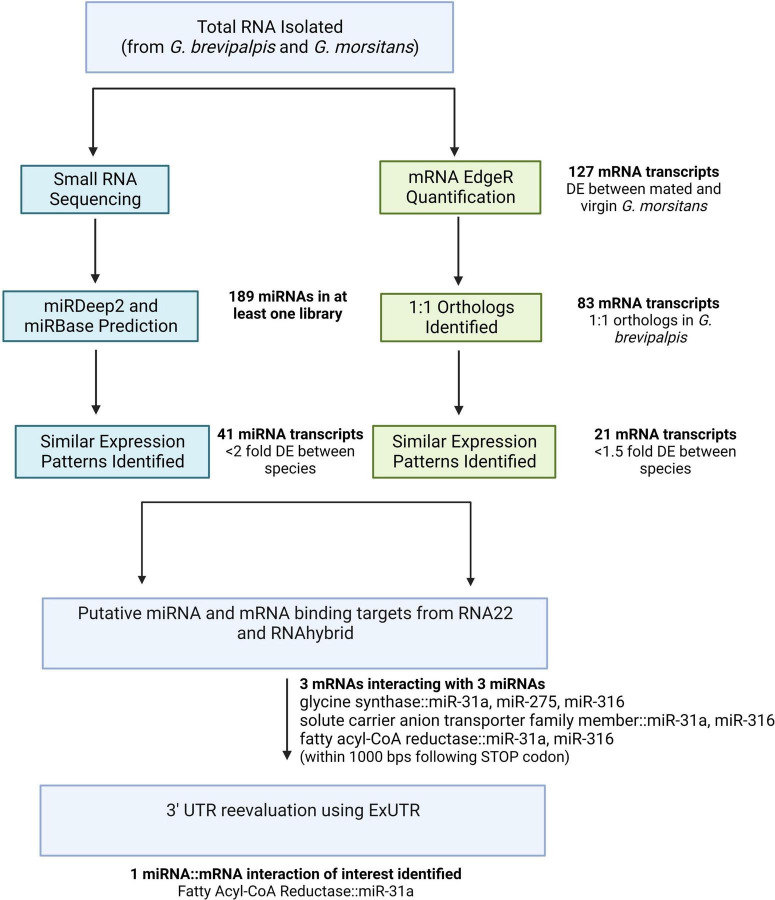
A simplified flowchart of the bioinformatic pipeline used to generate miRNA and mRNAs of interest. Three genes with multiple mRNA::miRNA interactions were predicted, with only miR-31a::fatty acyl CoA-reductase remaining following final evaluation with ExUTR. DE, differentially expressed.

Paired-end tags (PETs) from each RNA-Seq library were mapped to the corresponding reference genome of *G. morsitans* (GenBank: GCA_001077435.1) or *G. brevipalpis* (GenBank: GCA_000671755.1) by using subread v 2.0.1^2^ (Liao et al., 2013). Reads not mapping to the *Glossina* genomes were removed from further downstream analyses. To identify genes differentially expressed within the bacteriomes of both tsetse species relative to aposymbiotic tissue libraries, a custom bioinformatics pipeline was designed and implemented (Figure 1). Gene expression was quantified by RPKM (number of Reads Per Kilobase pairs of exon model per Million reads) taking the transcription annotation from VectorBase^3^ as a reference. Differential expression (DE) analyses comparing tissue, fly species and mating status were performed using edgeR (Empirical analysis of digital gene expression data in R). Significant differences in gene expression were determined with a false discovery rate of <0.05 and a fold-change of >1.5 (Robinson et al., 2010). Once genes of interest were identified in *G. morsitans*, only those with a known or 1:1 homolog predicted in VectorBase were examined in *G. brevipalpis.* Using Bioconductor, quantile normalization was applied to the libraries allowing for direct comparative analyses of gene expression between species (Bolstad et al., 2003). To understand the major putative functions for these genes of interest, eggNOG 5.0 was used to designate cluster of orthologous group categories (COGs) (Huerta-Cepas et al., 2016). High-quality (cutoff e-value <1.0e^–50^) assignments were given, if possible, with the remaining assigned based on the next best e-value.

### miRNA analyses

The miRNA-expressing genomic regions within tsetse tissues of interest were initially identified using miRDeep2 (default thresholds) which maps small RNAs to a reference genome and considers hairpin structures characteristic of miRNAs (Friedländer et al., 2008). Due to the selectivity of the software with a multiple-step read mapping process, the efficiency of miRDeep2 allows for consideration in both the sense and antisense directions and can exclude Argonaute-bound small RNAs lacking these characteristics features (Friedländer et al., 2008). Subsequently, precursor and mature miRNAs from miRbase were used as a reference to quantify the expression level of high confidence orthologs of the predicted tsetse miRNAs based on sequence identity (Figure 1; Griffiths-Jones et al., 2008).

### Predicting unique miRNAs

The miRbase software identifies miRNAs through comparison to a public repository of miRNA sequences with representation from 271 different species, though only 11% of the total miRNAs represented have an insect origin (Griffiths-Jones et al., 2008). These miRNA annotations are thus cataloged from other species, which inhibits the identification of unique miRNAs present in the tsetse fly. Some of these unique miRNAs may share seed regions with other miRNAs but originate from different precursors. Therefore, miRNAs identified by miRDeep2 based on structure and mapping to the genome, but lacking homologs within miRbase were analyzed to determine whether these may represent evolutionarily conserved unique *Glossinidae* miRNAs crucial to the ancient *Wigglesworthia* symbiosis. To determine the significance and likelihood of these miRNAs being present rather than artifacts of sequencing, we used a miRDeep2 threshold score of 4 (true positive rate ≥0.76) which is consistent with previous insect research (Feng et al., 2018). This provides a signal-to-noise ratio of∼50:1 in *G. brevipalpis* and∼30:1 in *G. morsitans*, respectively.

### Predicting miRNA::mRNA interactions

The 3′ UTRs of the mRNAs predicted to have conserved differential expression in the bacteriome of mated and virgin flies and between the bacteriome and the aposymbiotic tissue within the two tsetse species of interest were used to identify potential miRNA binding sites through RNAhybrid and RNA22 (Rehmsmeier et al., 2004; Krüger and Rehmsmeier, 2006). Initially, 3′ UTRs were defined as 1,000 bp downstream from the stop codon, as within related *Drosophila melanogaster*, 1,000 bp downstream of the stop codon encompasses >75% of all 3′ UTRs (Wang et al., 2019). Similarly, miRNAs showing conserved differential expression between bacteriomes and aposymbiotic tissue in both species were analyzed “*in silico”* for binding affinity as determined through free energy hybridization to the differentially expressed mRNAs using RNAhybrid and the pattern-based approach of RNA22. RNAhybrid v.2.1.2 analyses were performed with miRNA::mRNA binding energy <-14 kcal/mol and the helix constraint set from bases 2–7 with no preset p-value cutoff used (Sætrom et al., 2005). In RNA22 v. 2.0, the following settings were used: (a) sensitivity of 63, specificity of 61%, (b) seed size of 7, allow a maximum of 0 unpaired bases in seed region, (c) minimum number of paired-up bases in heteroduplex, 12, (d) maximum folding energy for heteroduplex (Kcal/mol), −12, and (e) maximum number of G:U wobbles allowed in seed region, 2.

After identifying putative miRNA::mRNA interactions, the bioinformatics pipeline was further developed to refine the UTR predictions from our transcriptome assemblies through the ExUTR package which predicts 3′ UTR regions after determining open reading frame (ORFs) from transcriptome data (Huang and Teeling, 2017). Bacteriome and aposymbiotic tissue mRNA libraries were assembled and mapped with HISAT2 (Kim et al., 2019)using the latest *Glossina* reference genomes FASTA files on Vectorbase (*G. morsitans* GmorY1 and *G. brevipalpis* GbreI1 VectorBase release 57) and selecting default parameters with option –dta-cufflinks. After conversion to BAM format and sorting and indexing with Samtools (Li et al., 2009), Cufflinks was used with default settings and provided the Vectorbase-release 57 gff files for each tsetse species used to assemble transcriptome files (Trapnell et al., 2010). For processing in ExUTR, FASTA files are required, however, an artifact of this pathway causes the multifasta generation from gff using GffRead to fail due to an issue with clipped bases, which was circumvented by employing the Cufftrim Perl script that truncates coordinates in a small number of sequences (Aechchiki, 2022). From transcripts of the mRNAs of interest, ORFs were identified through NCBI’s Open Reading Frame Finder and nucleotide BLAST (Wheeler et al., 2003). These translated ORFs could then serve as inputs for ExUTR 3′ UTR retrieval which was run with default settings (Huang and Teeling, 2017). Expression patterns at the 3′ UTRs of interest were further examined by using Integrated Genome Browser to identify paired reads with an average Phred score of >30 in UTR regions (Freese et al., 2016).

### Phylogenetic analyses

Genes sharing the same Enzyme Commission (EC) numbers as genes implicated in the miR-31a miRNA:: fatty acyl-CoA reductase mRNA interactions were compiled and amino acid sequences aligned using Clustal Omega for various insects (Sievers and Higgins, 2014). These genes were identified using a VectorBase search strategy that relied on Gene Ontology and the EC designation with additional homologs identified using BLASTp (Madden, 2002) or InsectBase 2.0 (Mei et al., 2022). Once aligned phylogenetic analyses were performed in a Maximum Likelihood framework using the IQ-TREE v. 1.6.12 webserver (Nguyen et al., 2015; Trifinopoulos et al., 2016) with the best model determined by ModelFinder (Kalyaanamoorthy et al., 2017). To assess phylogenetic robustness, 1,000 replicates of ultrafast bootstraps (UFB) (Hoang et al., 2018) and the SH-like approximate likelihood ratio test (SHaLRT) (Guindon et al., 2010) were performed. Clades with UFB≥95 and SHaLRT≥80 are considered to have strong support (Minh et al., 2021). Trees were visualized using the online tool available from the Interactive Tree of Life (iTOL) v5 (Letunic and Bork, 2021). Pseudogenization was predicted by visualization of the Pfam predicted domains using Simple Modular Architecture Research Tool (SMART) (Schultz et al., 2000; Letunic and Bork, 2018; Letunic et al., 2021) and NCBI’s Conserved Domain Database (CDD) (Marchler-Bauer and Bryant, 2004; Lu et al., 2019) with genes lacking one or more domains characteristic of fatty acyl-CoA reductases predicted to have a loss of function attributed to this gene family.

## Results

A total of 9 mRNA libraries were constructed from the bacteriomes or adjacent aposymbiotic tissues of age-matched *G. brevipalpis* or *G. morsitans* females of known mating status (Supplementary Table 1). We obtained an average of 19,409,921 ± 998,479 (Std. dev.) paired-end reads per library. As expected, many reads mapped back only to their corresponding genomes serving as a control for specificity (∼69%; *G. morsitans*; ∼75%; *G. brevipalpis*). A large portion of reads that did not map to tsetse stemmed from *Wigglesworthia* (data not shown). Consistently, the percentage of unmapped reads was significantly higher within the bacteriome (∼45%) compared to the aposymbiotic libraries (∼15%). Reads not mapping to the *Glossina* genomes were excluded from further downstream analyses.

**FIGURE 2:**
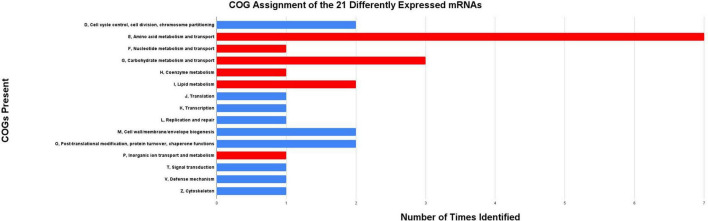
COGs assignments acquired from eggNOG 5.0. A total of 15 COG assignments are predicted for the 21 differentially expressed mRNAs between virgin and mated bacteriomes of *G. morsitans* with some mRNAs receiving multiple COG assignments. An enrichment of COG assignments in metabolism and transport functions (P, E, F, G, H, and I, noted with red bars) is observed for these mRNAs, with nearly half of these (47%) involved in amino acid metabolism and transport. S, Function unknown is not included.

An initial edgeR comparison identified a total of 127 differentially expressed mRNA transcripts between mated and virgin bacteriomes of *G. morsitans.* A total of 83 of these *G. morsitans* genes had a 1:1 homolog in *G. brevipalpis*, as identified through VectorBase. Using Bioconductor, quantile normalization was applied to the libraries allowing for direct comparative analyses of gene expression between species (Bolstad et al., 2003). A total of 21 of the 83 genes exhibited conserved interspecific expression patterns of <1.5-fold difference upon a comparison of *G. brevipalpis* and *G. morsitans* bacteriomes to their corresponding aposymbiotic libraries (Supplementary Table 2). To understand the major putative functions for these genes, eggNOG was used to designate cluster of orthologous group categories (COGs) (Huerta-Cepas et al., 2016). A large portion of COGs (*n* = 6 of 15 assignments, representing∼40% of the assigned COGs) was associated with metabolism and transport functions (Figure 2), concurring with the metabolic roles attributed to the bacteriomes (Snyder and Rio, 2015; Bing et al., 2017; Medina Munoz et al., 2017; Rio et al., 2019). Most genes (*n* = 17) had at least one high-quality COG assignment (Figure 2; Supplementary Table 2). Nearly half (46.7%) of these assignments have predicted roles in amino acid metabolism and transport which supports the significance of amino acid provisioning by either the tsetse fly or the blood diet to the mostly auxotrophic *Wigglesworthia* symbiont (i.e., incapable of synthesizing 15 of the 20 proteogenic amino acids). Except for the solute carrier organic anion transporter family member (GMOY004042/GBRI042772), all these genes were significantly upregulated in the bacteriomes of mated relative to virgin females (Supplementary Table 2).

### miRNA characterization

**FIGURE 3:**
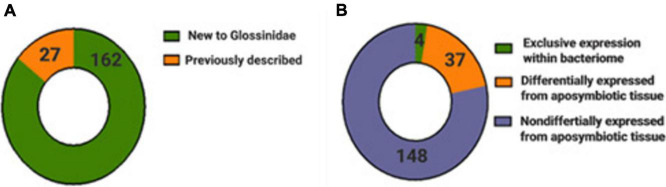
Donut charts for the miRNAs identified within tsetse bacteriome and aposymbiotic tissue. **(A)** The vast majority of identified miRNAs (86%) have not been previously documented in the Glossinidae family. **(B)** Most miRNAs identified were not differentially expressed between the bacteriome and aposymbiotic tissue (∼78%) while 22% showed differential expression. A total of 4 miRNAs (2%) had exclusive expression within bacteriomes. The 4 putative miRNAs considered to be unique to *Glossina* (1 in *G. morsitans* and 3 in *G. brevipalpis*) are not included in these numbers.

In both tsetse species, the raw small RNA read number in bacteriomes averaged ∼23,087,190 but was reduced to approximately ∼225,400 reads after filtering of low-quality reads, rRNA, and symbiont RNA (Supplementary Table 3). A total of 2,179 putative miRNAs were identified using miRDeep2 and the miRBase database to have expression in at least one bacteriome or aposymbiotic tissue library, which was then significantly collapsed to 395 miRNAs when homologs within seed families were eliminated. Additionally, low abundance miRNA transcripts were filtered out by removing miRNAs with lower than 10 raw reads in all libraries (Meki et al., 2018) resulting in 189 miRNAs with 66 of these miRNAs never described within Dipterans represented in miRbase (Supplementary Table 4). Further, when comparing the 189 miRNAs obtained in this study with the Glossinidae miRNA repository hosted on VectorBase, 162 miRNAs (∼86%) were identified as novel to the family (Figure 3). A total of 41 miRNAs were either under- or overexpressed by >2-fold RPKM upon the comparison of the small RNA libraries of the bacteriome to those of aposymbiotic tissue of mated flies with four of these miRNAs exclusively expressed within the bacteriome (Figure 3; Supplementary Table 5). Interestingly, one of the 189 predicted miRNAs, were observed only in one *Glossina* species. MiR-339 was identified only within a single *Glossina morsitans* library. Further, a total of one miRNA with no known homolog, thereby classified as “unique” was identified in *G. morsitans*, and three “unique” miRNAs were identified in *G. brevipalpis* of which none were shared either outside of *Glossina* or between species (Supplementary Table 6) precluding further investigation.

### Predicting miRNA::mRNA interactions

**FIGURE 4:**
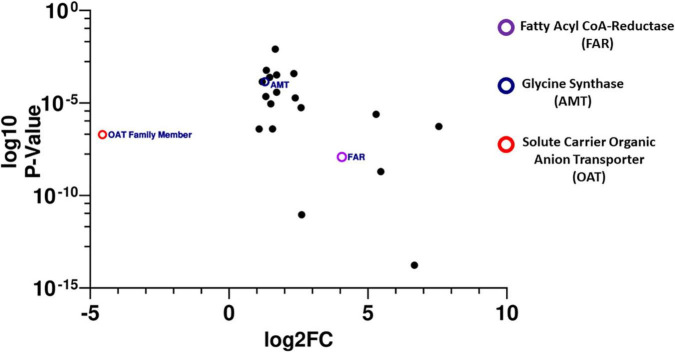
Plot showing the relative log2 fold change (x-axis) with significance (log10 adjusted *p*-value; *y*-axis) of the 21 mRNAs with differential expression between mated and virgin (mated/virgin) bacteriomes of *G. morsitans* and showing conserved expression patterns between *G. morsitans* and *G. brevipalpis* flies. Glycine synthase (AMT), a solute organic anion transporter family member (OAT), and fatty acyl-coA reductase (FAR) are predicted to be regulated by multiple miRNA interactions. The glycine synthase (*amt*) and fatty acyl-CoA genes (*far*) were significantly upregulated in the bacteriomes of mated relative to virgin females, while the solute carrier organic anion transporter family member (OAT) was downregulated.

**FIGURE 5:**
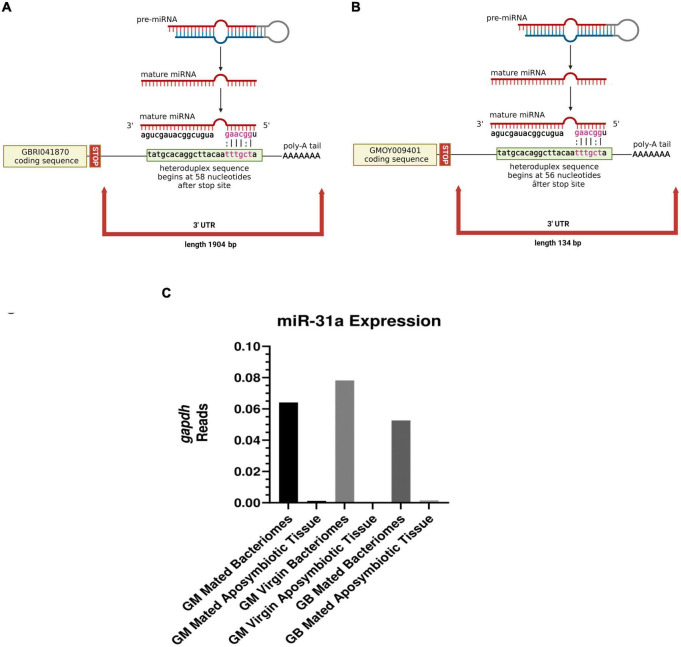
The predicted heteroduplex formations of fatty acyl-coA reductase in **(A)** *G. brevipalpis* (GBRI041870) and **(B)** *G. morsitans* (GMOY009401) and **(C)** the relative expression patterns of miR-31a to *gapDH* in the libraries examined. Nucleotides in pink represent the seed region and heteroduplex formation, | represents binding, and : represents a wobble.

A total of 26 miRNAs expressed within tsetse bacteriomes are predicted to interact with 19 of the 21 differentially expressed genes of interest in their 3′ UTR regions (Supplementary Table 7). A total of six of these miRNAs, (miR-11, miR-184b, miR-275, miR-316, miR-31a, and miR-956-5p) interacted with eight mRNAs in both species at a total of 13 predicted binding sites (Supplementary Figure 1). Of these eight genes, three have binding targets of multiple miRNAs, suggesting a potential importance to their regulation within the bacteriome. These genes were glycine synthase (*amt*) (GMOY001137/GBRI044528), fatty acyl-CoA reductase (*far*) (GMOY009401/GBRI041870), and a solute carrier organic anion transporter (OAT) family member (GMOY004042/GBRI042772) with strong sequence and protein similarity to OATP5A (Figure 4). The glycine synthase (*amt*) and fatty acyl-CoA (*far*) genes were significantly upregulated in the bacteriomes of mated relative to virgin females, while the solute carrier organic anion transporter family member (OAT) was downregulated (Figure 4). These putative miRNA::mRNA interactions were further examined by comparing the initial predictions of the 3′ UTR (i.e., 1,000 bp from STOP codon) with those predicted with ExUTR and VectorBase (Giraldo-Calderón et al., 2015; Huang and Teeling, 2017). The sole miRNA::mRNA interaction converged upon by these independent models was fatty acyl-CoA reductase (*far*) binding to miR-31a which is predicted to bind in approximately the same location past the STOP codon (58 nucleotides past STOP in *G. brevipalpis* and 56 nucleotides past STOP in *G. morsitans*) (Figures 5A, B). miR-31a has a significantly higher expression within bacteriomes relative to the aposymbiotic tissue in both tsetse species (30 fold-difference in *G. morsitans* and 104 fold-difference in *G. brevipalpis*) (Figure 5C).

### Phylogenetic analyses

**FIGURE 6:**
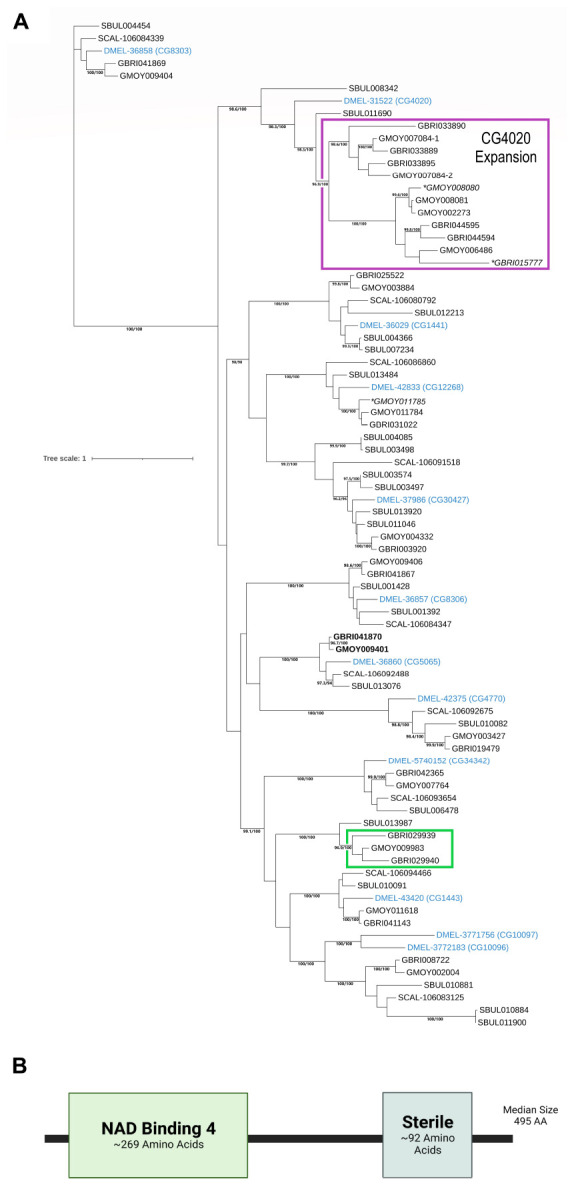
Phylogenetic and structural analyses of *Glossina* fatty acyl-CoA reductases. **(A)** A maximum likelihood tree for *Glossina* amino acid sequences identified as having fatty acyl-CoA activity by VectorBase in *Glossina morsitans* (*n* = 17) or *Glossina brevipalpis* (*n* = 18), genes identified as syntenic in *D. melanogaster and S. calcitrans*, and genes identified as *S. bullata* fatty acyl-CoA reductases in InsectBase v2. The LG+F+G4 substitution model was used. Genes are labeled by the first letter of genus name followed by first three letters of species name and the NCBI gene ID if available. For *Glossina* species or *S. bullata* this data is unavailable, therefore the respective VectorBase or InsectBase ID is used. Support values correspond to SHaLRT support (%)/ultrafast bootstrap support (%). Genes identified as pseudogenes or that may have acquired a novel function are italicized and indicated with a *. The *Glossina* fatty acyl-CoA genes predicted to interact with miR-31a are indicated in bold. The gene identified as GMOY007084 was identified as likely to be two genes, with the predicted sequences identified as –1 or –2, respectively, based on domain and revised STOP codon prediction. The scale bar corresponds to the number of substitutions per site. The *D. melanogaster* orthologs are indicated in blue, the CG4020 clade which indicates a *Glossina* specific gene expansion is boxed in purple, while the green box indicates GMOY009983, GBRI029940, and GBRI029939 which lack a *Drosophila* syntenic gene. **(B)** Representative Pfam domains predicted for fatty acyl-CoA reductase genes in *G. brevipalpis* and *G. morsitans* through SMART and NCBI CDD predictions.

The functional role(s) of *far* predicted by COG classification (i.e., lipid metabolism) was further characterized by identifying and analyzing orthologous and/or syntenic genes in VectorBase and those sharing an EC 1.2.1.84 designation (corresponding to alcohol-forming fatty acyl-CoA reductases). By examining the phylogenetic relationships of these genes, expansions, reductions, and the possibility of functional divergence within gene families may be deduced. VectorBase currently identifies three orthologs to GBRI041870 in *G. brevipalpis* and two to GMOY009401 in G. *morsitans.* As fatty acyl-CoA reductases are known to be much more abundant in closely related *Drosophila* species (Finet et al., 2019), VectorBase was searched for *G. morsitans* and *G. brevipalpis* genes classified with an EC number 1.2.1.84. A total of 21 and 23 genes had an EC number 1.2.1.84 assignment in the *G. morsitans* and *G. brevipalpis* genomes, respectively. Further examining whether synteny is preserved between these genes and those characterized as fatty acyl-CoA reductases within the *D. melanogaster* genome reveals that several of the predicted tsetse genes either had a higher UDP-glucuronosyltransferase EC prediction projected from Uniprot and/or were syntenic to a UDP-glucuronosyltransferase gene, therefore reducing the total number of fatty acyl-CoA reductase orthologs to 17 in *G. morsitans* and 18 in *G. brevipalpis* more closely resembling the number found in Drosophilidae members. Maximum likelihood analyses indicate that 12 fatty acyl-CoA reductases from *D. melanogaster* have orthologs in the two *Glossina* species with high support for these clades (Figure 6A). Notable gene family expansion is observed for *Glossina* species to the ortholog of *D. melanogaster* protein DMEL-31522 (CG4020). Sequences of the syntenic gene and additional genes within *Sarcophaga bullata* (flesh fly) identified as Fatty acyl-CoA reductase, along with the absence of an ortholog within *Stomoxys calcitrans* (stable fly), suggests that the expansion of CG4020 clade is unique within the Glossinidae rather than generalized to Calyptratae (Figure 6A).

As mentioned above, VectorBase identifies only GMOY009983/GBRI029939/GBRI029940, and GMOY011618/GBRI041143 as orthologous to GMOY009401/GBRI041870. GBRI041143 and GMOY011618 form a clade with CG1143, a gene known as *waterproof* (*wat*) in *D. melanogaster*, that reduces long chain fatty acid to fatty alcohols used in the hydrophobic inner tracheal lining and essential to gas exchange (Jaspers et al., 2014). GMOY009983, GBRI029940, and GBRI029939, interestingly, do clade together but appear to lack a syntenic gene to *D. melanogaster* and no ortholog in *S. calcitrans*, suggesting a FAR gene duplication event (Figure 6A). Of these three genes, two lack the Sterile domain (GBRI029940 and GBRI029939), and only GMOY009983 retains the two domains associated with FARs suggesting pseudogenization or the acquisition of a novel function in GBRI029940 and GBRI029939 (Figure 6B).

Our genes of interest that are likely regulated by miR-31a, GBRI041870 and GMOY00941, clade strongly with *D. melanogaster* CG5065 and preserve the domain architecture of most FARs by retaining NAD Binding 4 and Sterile domains (Figure 6B). Additional phylogenetic analyses including GMOY009401, GBRI041870 and syntenic orthologs within four other *Glossina* species (*G. austeni, G. fuscipes, G. pallidipes*, and *G. palpalis*), *D. melanogaster*, S. *calcitrans, Musca domestica, Aedes aegypti, Rhodnius prolixus, Pediculus humanus*, *Cimex lectularius* and a non-syntenic *Apis mellifera* protein previously identified as the *Apis* CG5065 (AMEL-100576414) (Teerawanichpan et al., 2010) further validate the clade of GMOY009401 and GBRI041870 within CG5065 (Supplementary Figure 2). Despite the previous characterization, the *A. mellifera* Far protein does not appear to be a member of CG5065 clade.

## Discussion

MiRNAs have emerged as potential regulators of homeostasis between insects and members of their microbiota (Aksoy et al., 2016; Feng et al., 2018, 2019; Mukherjee et al., 2020; Sun et al., 2022). Some miRNAs have been found to be associated with the bacteriomes in the aphid-*Buchnera* nutritional mutualism although the role of these miRNAs is still being evaluated (Feng et al., 2018, 2019). A miRNA, identified as novel-m0780-5p, in a similar obligate symbiosis in the white fly, *Bemisia tabaci*, impacts the horizontally transferred genes *panBC* involved in pantothenate (B5) production by reducing expression and impacting the titre of the *Portiera* symbiotic bacteria. MiRNAs are also known to play significant roles within various tsetse-microbial interactions. For example, the reduction of miR-275 upon trypanosome infection (or likewise the introduction of their surface coat Variant Surface Glycoprotein) disrupts the function of the peritrophic matrix (PM) by interfering with the regulation of the Wnt pathway and the Iroquois/IRX family of transcription factors (Aksoy et al., 2016; Yang et al., 2021). Trypanosomes do not harbor a chitinase enzyme within their genomes to degrade the PM, leading to the significance of the VSG, miR-275, and PM interaction toward facilitating trypanosome establishment. This PM disruption can be phenocopied either through the oral introduction of miRNA-275 antagomirs or through their production by genetically modified gut bacteria *Sodalis glossinidius* (Aksoy et al., 2016; Yang et al., 2021) within the tsetse midgut. Lastly, tsetse infections with asymptomatic (latent, no fitness cost) versus symptomatic Salivary Gland Hypertrophy Virus (GpSGHV; family Hytrosaviridae) display differences in their miRNA profiles (Meki et al., 2018) suggesting roles in the immunological interpretations of these viral infections.

It is likely that the extensive co-evolutionary history of tsetse and *Wigglesworthia*, dating back 50–80 million years, arose before Glossinidae radiation likely enabling the limited tsetse dietary ecology of vertebrate blood (Chen et al., 1999). In this study, total RNA was extracted from aposymbiotic and *Wigglesworthia*-containing organs in two distantly related species of tsetse flies. By comparing *G. brevipalpis*, an early diverging tsetse species (Krafsur, 2009; Attardo et al., 2019), to *G. morsitans*, a more recent species, it is likely that trends observed in mRNA::miRNA interactions within bacteriomes conserved across tsetse evolution may signify functional relevance toward the *Wigglesworthia*-tsetse symbiosis preserved through time. Differentially expressed RNAs within bacteriomes relative to aposymbiotic tissue are likely instrumental to the tsetse-*Wigglesworthia* symbiosis and may be regulated through epigenetic mechanisms including the action of miRNAs.

Ultimately, a single interaction between mir-31a and a putative fatty acyl-CoA reductase shows evidence of conservation in both tsetse species during pregnancy. Both mir-31a and fatty acyl-CoA reductase show increased expression in mated bacteriomes relative to the adjacent aposymbiotic tissue, and therefore may play a role in maintaining the *Wigglesworthia*-tsetse symbiosis. This fatty acyl-CoA reductase further shows increased mated bacteriome expression compared to virgin bacteriome expression. As miRNAs are usually involved in the downregulation of genes, this implies that a level of regulation of this gene is required, potentially toward scaling relative to increased gene expression in pregnant flies. Family members of miR-31 with identical seed regions have been studied in various insects. For example, mir-31-5p plays a role in cold tolerance in wood-boring beetles, while in *Drosophila* miR-31 is needed for proper gliogenesis in the adult brain, larval segmentation and has roles in the downregulation of the *wntless* (*wls*) regulator of the Wnt pathway. The Wnt pathway has diverse roles in cell fate determination, cell renewal, cell proliferation, embryonic development, and neural development (Bänziger et al., 2006).

Although fatty acyl-CoA reductase is characterized in many other insects including moths, *Drosophila*, and honeybees; the general function remains the same, to convert fatty-acyl precursors to alcohols which may serve as either pheromones or components of esters and lipids (Moto et al., 2003; Antony et al., 2009; Jaspers et al., 2014; Finet et al., 2019). The impact of odors on tsetse flies are well known, with olfactory attractants serving a vital role in trap-based tsetse control. Fatty acyl-CoA reductase-dependent insect cuticular hydrocarbons (CHCs), which function both as short-range recognition pheromones and to prevent desiccation, have already been explored for a link to the microbiota, with significantly higher expression of CHCs in wildtype compared to aposymbiotic tsetse flies (Engl et al., 2018; Finet et al., 2019).

Interestingly, *Glossina* has previously been characterized as highly reliant on tri- and diglycerides for energy, adapting to a reduction in carbohydrate metabolism to accommodate for its strict blood-feeding dietary ecology (International Glossina Genome Initiative, 2014). The phylogenetic evidence suggests that *Glossina* species have undergone multiple gene expansions within fatty acyl-CoA reductase genes, as was stated in the initial genome description (International Glossina Genome Initiative, 2014). Despite this, there is also a loss of multiple fatty acyl-CoA reductases characterized in *Drosophila melanogaster* (Finet et al., 2019). In contrast to concerted evolution, the fatty acyl-CoA reductase gene family is predicted to follow a “birth and death” model of evolution, in which duplications and subsequent diversification lead to gene family expansions (Ota and Nei, 1994; Finet et al., 2019). Here, despite duplications, the number of functional genes typically remains consistent with many duplications becoming nonfunctional and remaining only in a pseudogenized state or eventually purged from the genome following the accumulation of further deleterious mutations (Thomas, 2007). Furthermore, genes undergoing the “birth and death” model of evolution can be identified as “stable” or “unstable” with the former persisting as single copy genes over evolutionary distant organisms while the latter undergo frequent birth and death events leading to many duplications (Thomas, 2007). The duplicates also provide opportunities for novel functions to arise, separately or concurrently with the original function (Force et al., 1999).

In a previous study 17 FARs were identified in *D. melanogaster*, five of which were identified as unstable and 12 which were identified as stable across the *Drosophila* family (Finet et al., 2019). *Glossina* appears to have lost four of the five *D. melanogaster* genes identified as unstable (CG17562, CG14893, CG13091, and CG17560). In addition, the stable *Drosophila* gene *CG18031, fatty acyl-CoA reductase in oenocytes* (*farO*), which reduces very long chain fatty acids and is localized to and prevents overgrowth of larval oenocytes is seemingly lost in *Glossina* likely as an adaptation to “*in utero”* growth by tsetse larvae (Cinnamon et al., 2016; Finet et al., 2019). Unstable FARs have previously been assumed to be involved in CHC production with rapid evolution within *Drosophila* species enabling their diversification (Finet et al., 2019). Three of the unstable genes lost (CG17562, CG14893, and CG13091) and the one stable gene lost (CG18031, *farO*) within tsetse, are not lethal if disrupted in *Drosophila* larva as supported through RNAi knockdown (Finet et al., 2019). Interestingly, the last unstable gene apparently lost in *Glossina*, CG17560 results in a lethal phenotype in *Drosophila* larvae (Finet et al., 2019). In adult *D. melanogaster* and *D. serrata*, RNAi silencing of CG17560 leads to a reduction of short-chain CHCs resulting in increased long-chain CHC production and thereby influencing mating success (Bosco-Drayon et al., 2012). Pheromone production is an important role for CHCs, and some CHCs coordinate male sexual behavior in tsetse flies (Langley et al., 1975; Carlson et al., 1978, 1984; Huyton et al., 1980; Engl et al., 2018). Though pheromone and odorant research in tsetse remains in its infancy, the capacity for pheromone production by tsetse may be decreased relative to *Drosophila*. In support, the trichoid sensilla of the antennae of *G. morsitans* demonstrate decreased sensitivity to only the insect sex pheromone methyl laurate when six pheromones known from other dipterans were applied, though animal odorants, most significantly the alcohols 2-pentanol, and 1-hexen-3-ol, showed strong responses, demonstrating a functional shift toward odorant reception in general rather than the more pheromone focused trichoid sensilla in *Drosophila* (Soni et al., 2019).

In this study, the fatty acyl-CoA reductases GMOY009401 and GBRI041870 were identified as likely regulated by miR-31a. These genes are orthologs of the *Drosophila* gene CG5065, a gene classified as stable as it is required for successful larval development in *Drosophila* (Finet et al., 2019). RNAi analyses across the genome of *Drosophila* suggests that the product of CG5065 activity may be an epithelial cuticular hydrocarbon (CHC) as follicular epithelium development is disrupted following knockdown (Berns et al., 2014). The specialized nature of the tsetse bacteriome to exclusively house the obligate mutualist *Wigglesworthia* paired with the differential patterns of GMOY009401/GBRI041870 expression, however, suggest a role in mediating the symbiosis during mating. This may manifest in a role for this specific *far* gene toward the structural integrity of the bacteriome organ itself, which is itself composed of specialized epithelium cells. Tsetse reproduction (particularly given the adenotrophic viviparity reproductive mode) is metabolically expensive and necessitates increased nutrient provisioning from *Wigglesworthia* which may require developmental changes in the bacteriome for adjustment to these provisioning demands (Rio et al., 2006; Snyder and Rio, 2015).

These results establish a basis for future miRNA::mRNA research in the tsetse fly. With a putative miRNA 31a::fatty acyl-CoA reductase interaction within bacteriomes, experimental disruption of this binding may elucidate a more defined role in symbiosis. Antagomirs, antisense miRNAs which bind to the miRNA with greater affinity than the mRNA target due to specific and complementary design to the miRNA, are a good next step (Velu and Grimes, 2012) to uncover the biological significance of this interaction. Previous research has demonstrated that blood meal provided antagomirs can successfully downregulate miRNA expression in the tsetse fly (Aksoy et al., 2016). If this interaction is essential to the tsetse-*Wigglesworthia* symbiosis, phenotypic changes may be observed in important markers of fitness such as tsetse reproductive output, bacteriome integrity, and *Wigglesworthia* vertical transmission and population density. An abundant number of miRNAs and mRNAs were found to be differentially expressed in the bacteriome suggesting their importance in the symbiosis. Despite these findings, only a very small number of genes were initially predicted to be both differentially expressed in the bacteriome and targeted by differentially expressed miRNAs within two evolutionarily divergent tsetse species, supporting the utility of bioinformatic approaches to narrow targets for downstream experimental analyses. Ultimately, this line of research may aid in the development of novel tsetse control mechanisms which aim to disrupt the essential *Wigglesworthia* symbiosis resulting in tsetse sterility and population suppression.

## Data availability statement

The datasets presented in this study can be found in online repositories. The names of the repository/repositories and accession number(s) can be found in the article.

## Author contributions

ML and RR: experimental design, data collection, experimental analyses, and writing manuscript. GH: experimental analyses and writing manuscript. All authors contributed to the article and approved the submitted version.

## Footnotes

1
https://www.bioinformatics.babraham.ac.uk/projects/trim_galore/

2
http://subread.sourceforge.net/

3
https://vectorbase.org/vectorbase/app

## Supplementary material

Supplementary Figure 1 The six miRNAs (circles) predicted to interact with eight genes at their 3′ UTRs in both tsetse species following RNA22 and RNAHybrid analyses. Predicted miRNA::mRNA interactions are indicated with arrows.

Supplementary Figure 2 A maximum likelihood tree of amino acid sequences of genes predicted as orthologs to GMOY009401/GBRI041870, GMOY009983/GBRI029939/GBRI029940 and GMOY011618/GBRI041143 under the LG+F+G4 substitution model. Included are the syntenic orthologs from VectorBase including the corresponding *G. fuscipes, G. pallidipes, G. austeni*, *G. palpalis, D. melanogaster, M. domestica*, and *S. calcitrans* sequences. In addition, the NCBI sequences for the top ten BLASTP results for GMOY009401 and GBRI041870 (excluding *Glossina* species), and the *P. humanus*, *C. lectularius* and *A. mellifera* proteins are included. Genes are labeled by first letter of genus name followed by first three letters of species name then either the NCBI or VectorBase gene ID. In two cases the locus number is used for proteins that do not yet have a gene assignment (ZBOG-KAH8420979 and SBUL-TMW48389). Support values correspond to SHaLRT support (%)/ultrafast bootstrap support (%). Genes identified as pseudogenes or that may have acquired a novel function are italicized and indicated with a *. The genes predicted to interact with miR-31a are indicated in bold. The scale bar corresponds to the number of substitutions per site.

Supplementary Table 1 A summary of the mRNA libraries from *G. brevipalpis* mated females (bacteriomes; *n* = 2, aposymbiotic tissue; *n* = 1), *G. morsitans* mated females (bacteriomes; *n* = 2, aposymbiotic tissue; *n* = 1) and *G. morsitans* virgin females (bacteriome; *n* = 2, aposymbiotic tissue; *n* = 1).

Supplementary Table 2 A table of mRNAs with significant differential expression between *G. morsitans* mated and virgin bacteriomes and their designated COGs. For specific mRNAs in the absence of any COGs reaching the 1e^–50^ threshold, the next lowest COG is listed.

Supplementary Table 3 Summary of *G. morsitans* and *G. brevipalpis* small RNA libraries following quality filtering, rRNA removal, and bacterial RNA removal. miRDeep2 was used to map small RNA reads to respective tsetse species genomes. Numbers assigned to samples.

Supplementary Table 4 Predicted tsetse fly miRNAs showing expression of >10 raw reads in at least one library (*n* = 189). miRNA designations not previously observed within Diptera represented in miRBase are shown in red (*n* = 66).

Supplementary Table 5 Predicted tsetse fly miRNAs showing conserved expression patterns of either over expression (>2-fold DE) or under expression (<2-fold DE) in bacteriomes relative to aposymbiotic tissue. Seed regions are shown in red. Those miRNAs exclusively expressed in the bacteriomes are presented in blue rows (*n* = 4), and miR-31a indicated in orange.

Supplementary Table 6 Predicted novel microRNAs in *G. morsitans* (*n* = 1) and *G. brevipalpis* (*n* = 3).

Supplementary Table 7 Predicted 3′ UTR interactions between differentially expressed miRNAs and mRNAs within bacteriomes in at least one of the tsetse species.

## References

[B1] Aechchiki (2022). *rna seq – gffread: GFaSeqGet errors on coordinate overhang – Bioinformatics Stack Exchange.* Available online at: https://bioinformatics.stackexchange.com/questions/3651/gffread-gfaseqget-errors-on-coordinate-overhang (accessed June 20, 2022).

[B2] Aksoy E. Vigneron A. Bing X. Zhaoa X. O’Neill M. Wu Y. (2016). Mammalian African trypanosome VSG coat enhances tsetse’s vector competence. *Proc. Natl. Acad. Sci. U.S.A.* 113 6961–6966. 10.1073/pnas.1600304113 27185908 PMC4922192

[B3] Aksoy S. (1995). *Wigglesworthia* gen. nov. and *Wigglesworthia glossinidia* sp. nov., taxa consisting of the mycetocyte-associated, primary endosymbionts of tsetse flies. *Int. Syst. Bacteriol.* 45 848–851. 10.1099/00207713-45-4-848 7547309

[B4] Antony B. Fujii T. Moto K. Matsumoto S. Fukuzawa M. Nakano R. (2009). Pheromone-gland-specific fatty-acyl reductase in the adzuki bean borer, *Ostrinia scapulalis* (Lepidoptera: Crambidae). *Insect Biochem. Mol. Biol.* 39 90–95. 10.1016/j.ibmb.2008.10.008 19041942

[B5] Attardo G. Abd-Alla A. Acosta-Serrano A. Allen J. Bateta R. Benoit J. (2019). Comparative genomic analysis of six Glossina genomes, vectors of African trypanosomes. *Genome Biol.* 20:187. 10.1186/s13059-019-1768-2 PMC6721284 31477173

[B6] Bänziger C. Soldini D. Schütt C. Zipperlen P. Hausmann G. Basler K. (2006). Wntless, a conserved membrane protein dedicated to the secretion of Wnt proteins from signaling cells. *Cell* 125 509–522. 10.1016/j.cell.2006.02.049 16678095

[B7] Berns N. Woichansky I. Friedrichsen S. Kraft N. Riechmann V. (2014). A genome-scale in vivo RNAi analysis of epithelial development in *Drosophila* identifies new proliferation domains outside of the stem cell niche. *J. Cell Sci.* 127 2736–2748. 10.1242/jcs.144519 24762813

[B8] Bing X. Attardo G. Vigneron A. Aksoy E. Scolari F. Malacrida A. (2017). Unravelling the relationship between the tsetse fly and its obligate symbiont Wigglesworthia: Transcriptomic and metabolomic landscapes reveal highly integrated physiological networks. *Proc. R. Soc. B* 284:20170360. 10.1098/rspb.2017.0360 28659447 PMC5489720

[B9] Bolstad B. Irizarry R. Astrand M. Speed T. (2003). A comparison of normalization methods for high density oligonucleotide array data based on variance and bias. *Bioinformatics (Oxford, England)* 19 185–193. 12538238 10.1093/bioinformatics/19.2.185

[B10] Bosco-Drayon V. Poidevin M. Boneca I. Narbonne-Reveau K. Royet J. Charroux B. (2012). Peptidoglycan sensing by the receptor PGRP-LE in the Drosophila gut induces immune responses to infectious bacteria and tolerance to microbiota. *Cell Host Microbe* 12 153–165. 10.1016/j.chom.2012.06.002 22901536

[B11] Carlson D. Langley P. Huyton P. (1978). Sex pheromone of the tsetse fly: Isolation, identification, and synthesis of contact aphrodisiacs. *Science (New York, N.Y.)* 201 750–753. 10.1126/science.675256 675256

[B12] Carlson D. Nelson D. Langley P. Coates T. Davis T. Leegwater-Van Der Linden M. (1984). Contact sex pheromone in the tsetse fly *Glossina pallidipes* (Austen) Identification and Synthesis. *J. Chem. Ecol.* 10 429–450. 10.1007/BF00988090 24318549

[B13] Chen X. Li S. Aksoy S. (1999). Concordant evolution of a symbiont with its host insect species: Molecular phylogeny of genus *Glossina* and its bacteriome-associated endosymbiont, *Wigglesworthia glossinidia*. *J. Mol. Evol.* 48 49–58. 10.1007/pl00006444 9873076

[B14] Cinnamon E. Makki R. Sawala A. Wickenberg L. Blomquist G. Tittiger C. (2016). Drosophila spidey/Kar regulates oenocyte growth via PI3-kinase signaling. *PLoS Genet.* 12:1006154. 10.1371/journal.pgen.1006154 27500738 PMC4976899

[B15] Cross G. (1978). Antigenic variation in trypanosomes. *Proc. R. Soc. Lond. Biol. Sci.* 202 55–72. 10.1098/RSPB.1978.0057 27816

[B16] Engl T. Michalkova V. Weiss B. Uzel G. Takac P. Miller W. (2018). Effect of antibiotic treatment and gamma-irradiation on cuticular hydrocarbon profiles and mate choice in tsetse flies (Glossina m. morsitans). *BMC Microbiol.* 18 155–167. 10.1186/s12866-018-1292-7 30470188 PMC6251160

[B17] Feng H. Park J. Zhai R. Wilson A. (2019). MicroRNA-92a regulates the expression of aphid bacteriocyte-specific secreted protein 1. *BMC Res. Notes* 12:638. 10.1186/s13104-019-4665-6 31564246 PMC6767646

[B18] Feng H. Wang L. Wuchty S. Wilson A. (2018). microRNA regulation in an ancient obligate endosymbiosis. *Mol. Ecol.* 27 1777–1793. 10.1111/mec.14464 29271121

[B19] Finet C. Slavik K. Pu J. Carroll S. Chung H. (2019). Birth-and-death evolution of the fatty Acyl-CoA reductase (FAR) gene family and diversification of cuticular hydrocarbon synthesis in *Drosophila*. *Genome Biol. Evol.* 11:1541. 10.1093/gbe/evz094 31076758 PMC6546124

[B20] Force A. Lynch M. Pickett F. Amores A. Yan Y. Postlethwait J. (1999). Preservation of duplicate genes by complementary, degenerative mutations. *Genetics* 151:1531. 10.1093/genetics/151.4.1531 PMC1460548 10101175

[B21] Freese N. Norris D. Loraine A. (2016). Integrated genome browser: Visual analytics platform for genomics. *Bioinformatics (Oxford, England).* 32 2089–2095. 27153568 10.1093/bioinformatics/btw069 PMC4937187

[B22] Friedländer M. Chen W. Adamidi C. Maaskola J. Einspanier R. Knespel S. (2008). Discovering microRNAs from deep sequencing data using miRDeep. *Nat. Biotechnol.* 26 407–415. 18392026 10.1038/nbt1394

[B23] Giraldo-Calderón G. Emrich S. MacCallum R. Maslen G. Dialynas E. Topalis P. (2015). VectorBase: an updated bioinformatics resource for invertebrate vectors and other organisms related with human diseases. *Nucleic Acids Res.* 43 D707–D713. 10.1093/nar/gku1117 25510499 PMC4383932

[B24] Griffiths-Jones S. Saini H. Van Dongen S. Enright A. (2008). miRBase: Tools for microRNA genomics. *Nucleic Acids Res.* 36(suppl_1) D154–D158. 17991681 10.1093/nar/gkm952 PMC2238936

[B25] Guindon S. Dufayard J. Lefort V. Anisimova M. Hordijk W. Gascuel O. (2010). New algorithms and methods to estimate maximum-likelihood phylogenies: Assessing the performance of PhyML 3.0. *Syst. Biol.* 59 307–321. 10.1093/sysbio/syq010 20525638

[B26] Hoang D. Chernomor O. Von Haeseler A. Minh B. Vinh L. (2018). UFBoot2: Improving the ultrafast bootstrap approximation. *Mol. Biol. Evol.* 35 518–522. 10.1093/molbev/msx281 29077904 PMC5850222

[B27] Huang Z. Teeling E. (2017). ExUTR: A novel pipeline for large-scale prediction of 3’-UTR sequences from NGS data. *BMC Genomics* 18:847. 10.1186/s12864-017-4241-1 29110697 PMC5674806

[B28] Huerta-Cepas J. Szklarczyk D. Forslund K. Cook H. Heller D. Walter M. (2016). eggNOG 4.5: A hierarchical orthology framework with improved functional annotations for eukaryotic, prokaryotic and viral sequences. *Nucleic Acids Res.* 44 D286–D293. 10.1093/nar/gkv1248 26582926 PMC4702882

[B29] Huyton P. Langley P. Carlson D. Schwarz M. (1980). Specificity of contact sex pheromones in tsetse flies, *Glossina* spp. *Physiol. Entomol.* 5 253–264.

[B30] International Glossina Genome Initiative (2014). Genome sequence of the tsetse fly (*Glossina morsitans*): Vector of African trypanosomiasis. *Science* 344 380–386. 10.1126/science.1249656 24763584 PMC4077534

[B31] Jaspers M. Pflanz R. Riedel D. Kawelke S. Feussner I. Schuh R. (2014). The fatty acyl-CoA reductase Waterproof mediates airway clearance in *Drosophila*. *Dev. Biol.* 385 23–31. 10.1016/j.ydbio.2013.10.022 24183938

[B32] Kalyaanamoorthy S. Minh B. Wong T. Von Haeseler A. Jermiin L. (2017). ModelFinder: fast model selection for accurate phylogenetic estimates. *Nat. Methods* 14 587–589. 10.1038/nmeth.4285 28481363 PMC5453245

[B33] Kim D. Paggi J. Park C. Bennett C. Salzberg S. (2019). Graph-based genome alignment and genotyping with HISAT2 and HISAT-genotype. *Nat. Biotechnol.* 37 907–915. 31375807 10.1038/s41587-019-0201-4 PMC7605509

[B34] Krafsur E. (2009). Tsetse flies: Genetics, evolution, and role as vectors. *Infect. Genet. Evol.* 9 124. 10.1016/j.meegid.2008.09.010 PMC2652644 18992846

[B35] Krüger J. Rehmsmeier M. (2006). RNAhybrid: microRNA target prediction easy, fast and flexible. *Nucleic Acids Res.* 34 W451–W454. 10.1093/nar/gkl243 16845047 PMC1538877

[B36] Langley P. Pimley R. Carlson D. (1975). Sex recognition pheromone in tsetse fly *Glossina morsitans*. *Nature.* 254 51–53. 10.1038/254051a0 1113875

[B37] Letunic I. Bork P. (2018). 20 years of the SMART protein domain annotation resource. *Nucleic Acids Res.* 46 D493–D496. 10.1093/nar/gkx922 29040681 PMC5753352

[B38] Letunic I. Bork P. (2021). Interactive Tree Of Life (iTOL) v5: an online tool for phylogenetic tree display and annotation. *Nucleic Acids Res.* 49 W293–W296. 10.1093/nar/gkab301 33885785 PMC8265157

[B39] Letunic I. Khedkar S. Bork P. (2021). SMART: Recent updates, new developments and status in 2020. *Nucleic Acids Res.* 49 D458–D460. 10.1093/nar/gkaa937 33104802 PMC7778883

[B40] Li H. Handsaker B. Wysoker A. Fennell T. Ruan J. Homer N. (2009). The sequence alignment/map format and SAMtools. *Bioinformatics* 25 2078–2079. 10.1093/bioinformatics/btp352 19505943 PMC2723002

[B41] Liao Y. Smyth G. Shi W. (2013). The Subread aligner: fast, accurate and scalable read mapping by seed-and-vote. *Nucleic Acids Res.* 41:e108. 10.1093/nar/gkt214 23558742 PMC3664803

[B42] Lu S. Wang J. Chitsaz F. Derbyshire M. Geer R. Gonzales N. (2019). CDD/SPARCLE: the conserved domain database in 2020. *Nucleic Acids Res.* 48 D265–D268. 10.1093/nar/gkz991 31777944 PMC6943070

[B43] Madden T. (2002). “The BLAST sequence analysis tool,” in *The NCBI handbook*, eds. McEntyre J. Ostell J. (Bethesda, MD: National Center for Biotechnology Information (US)).

[B44] Marchler-Bauer A. Bryant S. H. C. D. - (2004). Search: Protein domain annotations on the fly. *Nucleic Acids Res.* 32 W327–W331. 10.1093/nar/gkh454 15215404 PMC441592

[B45] Medina Munoz M. Pollio A. White H. Rio R. Munoz M. Pollio A. (2017). Into the wild: parallel transcriptomics of the tsetse-wigglesworthia mutualism within kenyan populations. *Genome Biol. Evol.* 9 2276–2291. 10.1093/gbe/evx175 28934375 PMC5601960

[B46] Mei Y. Jing D. Tang S. Chen X. Chen H. Duanmu H. (2022). InsectBase 2.0: A comprehensive gene resource for insects. *Nucleic Acids Res.* 50 D1040–D1045. 10.1093/nar/gkab1090 34792158 PMC8728184

[B47] Meki I. Ince I. Kariithi H. Boucias D. Ozcan O. Parker A. (2018). Expression profile of *Glossina pallidipes* MicroRNAs during symptomatic and asymptomatic infection with *Glossina pallidipes* salivary gland hypertrophy virus (Hytrosavirus). *Front. Microbiol.* 9:2037. 10.3389/fmicb.2018.020 PMC6129597 30233523

[B48] Michalkova V. Benoit J. Weiss B. Attardo G. Aksoy S. (2014). Vitamin B6 generated by obligate symbionts is critical for maintaining proline homeostasis and fecundity in tsetse flies. *Appl. Environ. Microbiol.* 80 5844–5853. 10.1128/AEM.01150-14 25038091 PMC4178588

[B49] Minh B. Lanfear R. Ly N. Trifinopoulos J. Schrempf D. Schmidt H. (2021). *IQ-TREE Version 2.1. 2: Tutorials and manual phylogenomic software by maximum likelihood.* Available online at: http://www.iqtree.org/doc/iqtree-doc.pdf

[B50] Moloo S. (1971). An artificial feeding technique for glossina. *Parasitology* 63 507–512. 5139030 10.1017/s0031182000080021

[B51] Moto K. Yoshiga T. Yamamoto M. Takahashi S. Okano K. Ando T. (2003). Pheromone gland-specific fatty-acyl reductase of the silkmoth, Bombyx mori. *Proc. Natl. Acad. Sci.U.S.A.* 100 9156–9161. 12871998 10.1073/pnas.1531993100 PMC170888

[B52] Mukherjee K. Amsel D. Kalsy M. Billion A. Dobrindt U. Vilcinskas A. (2020). MicroRNAs regulate innate immunity against uropathogenic and commensal-like *Escherichia coli* infections in the surrogate insect model *Galleria mellonella*. *Sci. Rep.* 10:2570. 10.1038/s41598-020-59407-3 32054914 PMC7018962

[B53] Nguyen L. Schmidt H. Von Haeseler A. Minh B. (2015). IQ-TREE: A fast and effective stochastic algorithm for estimating maximum-likelihood phylogenies. *Mol. Biol. Evol.* 32 268–274. 10.1093/molbev/msu300 25371430 PMC4271533

[B54] Nogge G. (1976). Sterility in tsetse flies (*Glossina morsitans* Westwood) caused by loss of symbionts. *Experientia* 32 995–996. 10.1007/BF01933932 986317

[B55] Ota T. Nei M. (1994). Divergent evolution and evolution by the birth-and-death process in the immunoglobulin VH gene family. *Mol. Biol. Evol.* 11 469–482. 10.1093/oxfordjournals.molbev.a040127 8015440

[B56] Rehmsmeier M. Steffen P. Höchsmann M. Giegerich R. (2004). Fast and effective prediction of microRNA/target duplexes. *RNA* 10 1507–1517. 10.1261/rna.5248604 15383676 PMC1370637

[B57] Rio R. Jozwick A. Savage A. Sabet A. Vigneron A. Wu Y. (2019). Mutualist-provisioned resources impact vector competency. *mBio* 10:e00018–e19. 31164458 10.1128/mBio.00018-19 PMC6550517

[B58] Rio R. Wu Y. Filardo G. Aksoy S. (2006). Dynamics of multiple symbiont density regulation during host development: Tsetse fly and its microbial flora. *Proc. R. Soc. B* 273 805–814. 10.1098/rspb.2005.3399 16618673 PMC1560226

[B59] Robinson M. McCarthy D. Smyth G. (2010). edgeR: A Bioconductor package for differential expression analysis of digital gene expression data. *Bioinformatics* 26 139–140. 19910308 10.1093/bioinformatics/btp616 PMC2796818

[B60] Sætrom O. Snøve O. Sætrom P. (2005). Weighted sequence motifs as an improved seeding step in microRNA target prediction algorithms. *RNA* 11 995–1003. 10.1261/rna.7290705 15928346 PMC1370784

[B61] Schultz J. Copley R. Doerks T. Ponting C. Bork P. (2000). SMART: a web-based tool for the study of genetically mobile domains. *Nucleic Acids Res.* 28 231–234. 10.1093/nar/28.1.231 10592234 PMC102444

[B62] Sievers F. Higgins D. (2014). Clustal Omega, accurate alignment of very large numbers of sequences. *Methods Mol. Biol. (Clifton, N.J.).* 1079 105–116. 10.1007/978-1-62703-646-7_6 24170397

[B63] Snyder A. Rio R. (2015). “Wigglesworthia morsitans” folate (vitamin B9) biosynthesis contributes to tsetse host fitness. *Appl. Environ. Microbiol.* 81 5375–5386. 10.1128/AEM.00553-15 26025907 PMC4510189

[B64] Song J. Smith S. Hannon G. Joshua-Tor L. (2004). Crystal structure of Argonaute and its implications for RISC slicer activity. *Science (New York, N.Y.)* 305 1434–1437. 10.1126/science.1102514 15284453

[B65] Soni N. Sebastian Chahda J. Carlson J. (2019). Odor coding in the antenna of the tsetse fly *Glossina morsitans*. *Proc. Natl. Acad. Sci. U.S.A.* 116 14300–14308. 10.1073/pnas.1907075116 31221757 PMC6628836

[B66] Sun X. Liu B. Li C. Chen Z. Xu X. Luan J. (2022). A novel microRNA regulates cooperation between symbiont and a laterally acquired gene in the regulation of pantothenate biosynthesis within *Bemisia tabaci* whiteflies. *Mol. Ecol.* 31 2611–2624. 10.1111/mec.16416 35243711

[B67] Teerawanichpan P. Robertson A. Qiu X. (2010). A fatty acyl-CoA reductase highly expressed in the head of honey bee (Apis mellifera) involves biosynthesis of a wide range of aliphatic fatty alcohols. *Insect Biochem. Mol. Biol.* 40 641–649. 10.1016/J.IBMB.2010.06.004 20542116

[B68] Thomas J. (2007). Rapid birth–death evolution specific to xenobiotic cytochrome P450 genes in vertebrates Trask BJ, editor. *PLoS Genet.* 3:e67. 10.1371/journal.pgen.0030067 17500592 PMC1866355

[B69] Trapnell C. Williams B. Pertea G. Mortazavi A. Kwan G. Van Baren M. (2010). Transcript assembly and quantification by RNA-Seq reveals unannotated transcripts and isoform switching during cell differentiation. *Nat. Biotechnol.* 28 511–515. 10.1038/nbt.1621 20436464 PMC3146043

[B70] Trifinopoulos J. Nguyen L. von Haeseler A. Minh B. (2016). W-IQ-TREE: A fast online phylogenetic tool for maximum likelihood analysis. *Nucleic Acids Res.* 44 W232–W235. 10.1093/nar/gkw256 27084950 PMC4987875

[B71] Velu C. Grimes H. (2012). Utilizing AntagomiR (Antisense microRNA) to knock down microRNA in murine bone marrow cells. *Methods Mol. Biol. (Clifton, N.J.)* 928 185–195. 10.1007/978-1-62703-008-3_15 22956143 PMC4025929

[B72] Wang W. Fang D. Gan J. Shi Y. Tang H. Wang H. (2019). Evolutionary and functional implications of 3’ untranslated region length of mRNAs by comprehensive investigation among four taxonomically diverse metazoan species. *Genes Genomics.* 41 747–755. 10.1007/s13258-019-00808-8 30900191 PMC6560010

[B73] Weiss B. Maltz M. Aksoy S. (2012). Obligate symbionts activate immune system development in the tsetse fly. *J. Immunol.* 188 3395–3403. 22368278 10.4049/jimmunol.1103691 PMC3311772

[B74] Wheeler D. Church D. Federhen S. Lash A. Madden T. Pontius J. (2003). Database resources of the national center for biotechnology. *Nucleic Acids Res.* 31:28. 10.1093/nar/gkg033 PMC165480 12519941

[B75] Yang L. Weiss B. Williams A. Aksoy E. de Silva Orfano A. Son J. (2021). Paratransgenic manipulation of a tsetse microRNA alters the physiological homeostasis of the fly’s midgut environment. *PLoS Pathog.* 17:e1009475. 10.1371/journal.ppat.1009475 34107000 PMC8216540

[B76] Zhang X. Zheng Y. Cao X. Ren R. Yu X. Jiang H. (2015). Identification and profiling of Manduca sexta microRNAs andtheir possible roles in regulating specific transcripts in fat body, hemocytes, and midgut. *Insect Biochem. Mol. Biol.* 62:11. 25196249 10.1016/j.ibmb.2014.08.006 PMC4362813

