# Supplementary material for: Symbiosis preservation: Putative regulation of fatty acyl-CoA reductase by miR-31a within the symbiont harboring bacteriome through tsetse evolution
Source: Front Microbiol. 2023 Apr 11;14:1151319. doi: 10.3389/fmicb.2023.1151319 (PMC10126493; doi:10.3389/fmicb.2023.1151319)
Supplement: Supplementary Figure 1 — The six miRNAs (circles) predicted to interact with eight genes at their 3′ UTRs in both tsetse species following RNA22 and RNAHybrid analyses. Predicted miRNA::mRNA interactions are indicated with arrows. [file Data_Sheet_1.pdf]

S. Table 1

mRNA libraries	Average paired-end reads (± Std. dev.)	% mapping to *Glossina* genome
Glossina brevipalpis		
(2) female mated bacteriomes	19,711,604 ± 2,539,609	68%
(1) female mated crops and proventriculus	18,257,185	81%
Glossina morsitans		
(2) female mated bacteriome	19,930,775 ± 491,931	52%
(1) female mated crops and proventriculus	17,548,002	89%
(2) female virgin bacteriome	19,245,787 ± 233,733	44%
(1) female virgin crops and proventriculus	19,955,037	89%

S. Table 2

Gm Gene ID	Gb Gene ID	Gene name	COG classification	Major Domain(s)	Fold Change GM Mated/Virgin Bacteriomes
GMOY009401	GBRI041870	Fatty acyl-CoA reductase	I	fatty acyl-CoA reductase	Increase
GMOY006101	GBRI004801	dendritic arbor reduction 1	S, K	zinc finger C2H2-type	Increase
GMOY010240	GBRI003961	endoplasmic reticulum metallopeptidase 1	E, D, Z,	peptidase M28	Increase
GMOY000698	GBRI022302	aminopeptidase	O, E, J, I, V, D	aminopeptidase N-like, N-terminal	Increase
GMOY013248	GBRI005135	spermidine synthase	E	spermidine synthase	Increase
GMOY010090	GBRI030396	AICAR transformylase	F	AICAR transformylase, duplicated domain superfamily	Increase
GMOY009161	GBRI028525	chitinase-like protein Idgf5	G	chitinase II	Increase
GMOY005527	GBRI019337	pugilist	F, H,	major facilitator superfamily	Increase
GMOY006765	GBRI007097	sarcocystatin-A-like	S*	none (via SMART, UNIPROT)	Increase
GMOY005029	GBRI012464	serine protease 11	O, M, E, T	trypsin-like serine protease	Increase
GMOY001469	GBRI019785	Major Facilitator Superfamily Transporter or picot	G, L,	none (via SMART, UNIPROT)	Increase
GMOY004042	GBRI042772	solute carrier organic anion transporter family member	P	KAZAL	Decrease

GMOY003408	GBRI023418	hi	E	betaine-homocysteine S-methyltransferase, BHMT	Increase
GMOY000180	GBRI014796	TPR repeat-containing protein	T*	tetratricopeptide repeat-containing domain	Increase
GMOY012057	GBRI042754	organic cation transporter protein	S, E, M, G, T,	none (via SMART, UNIPROT)	Increase
GMOY001137	GBRI044528	glycine synthase	E, S	glycine cleavage system T protein	Increase
GMOY008996	GBRI040391	cuticular protein 49Ae	S*	none (via SMART, UNIPROT)	Increase
GMOY009120	GBRI028908	patched-related	G, S,	protein patched/dispatched	Increase
GMOY007451	GBRI043918	endocuticle structural glycoprotein	S*	none (via SMART, UNIPROT)	Increase
GMOY010676	GBRI002237	cuticular protein 57A	S	chitin-binding type R&R domain profile	Increase
GMOY000069	GBRI041971	unknown	S	PAN/apple domain	Increase

*no COG reached the 1e-50 threshold, the highest of e-value of >1e-50 was taken

A total of 36 individual COG designations fall within 16 categories.

COGS Present	Name	Number of Times Identified
D	Cell cycle control, cell division, chromosome partitioning	2
E	Amino Acid metabolism and transport	7
F	Nucleotide metabolism and transport	1
G	Carbohydrate metabolism and transport	3
H	Coenzyme metabolism	1
I	Lipid Metabolism	2
J	Translation	1
K	Transcription	1
L	Replication and Repair	1
M	Cell wall/membrane/envelop biogenesis	2
O	Post-translational modification, protein turnover, chaperone functions	2
P	Inorganic ion transport and metabolism	1
S	Function Unknown	9
T	Signal Transduction	1
V	Defense Mechanism	1
Z	Cytoskeleton	1

S. Table 3

Class of RNA	*Glossina brevipalpis* mated female bacteriome library 3	*Glossina brevipalpis* mated female bacteriome library 2
Raw Reads	24,644,079	21,630,810
Reads After Filter Quality	23,823,263	21,246,637
After Removing Fly rRNA	2,711,227	1,238,989
After Removing Symbiont RNA	822,619	417,839
Reads Mapping to Reference Genome	344,509	141,002
% Filtered Reads Mapping to Reference Genome	41.9%	33.7%

Class of RNA	*Glossina brevipalpis* mated female crop and proventriculus library 2	*Glossina morsitans* mated female crop and proventriculus library 1
Raw Reads	28,078,486	20,000,469
Reads After Filter Quality	27,802,904	19,859,113
After Removing Fly rRNA	487,987	764,898
After Removing Symbiont RNA	479,025	749,304
Reads Mapping to Reference Genome	207,023	370,376
% Filtered Reads Mapping to Reference Genome	43.2%	49.4%

Glossina morsitans mated female bacteriome library 2	*Glossina morsitans* mated female bacteriome library 3	*Glossina morsitans* virgin female bacteriome library 1
27,463,266	30,711,696	19,439,601
26,617,051	29,218,590	18,815,565
4,170,019	2,763,762	3,139,727
835,636	475,632	547,208
244,968	138,826	167,064
29.3%	29.2%	30.5%

Glossina morsitans virgin female crop and proventriculus library 3
24,493,884
24,190,483
564,100
548,042
236,867
43.2%

Glossina morsitans virgin female bacteriome library 3
14,633,686
14,010,508
6,646,610
1,066,487
316,028
29.6%

S. Table 4

miRNA	Mature miRNA
bantam	ugagaucauuuugaaagcugauu
bantam-3p	ugagaucauuuugaaagcugauu
bantam-5p	ccgguuuucgauuugguuugacu
let-7	ugagguaguagguuguauagu
let-7-5p	ugagguaguagguuguauagu
let-7a	ugagguaguagguuguauag
let-7a-5p	ugagguaguagguuguauaguu
let-7c	ugagguaguagguuguaugguu
let-7c-5p	ugagguaguagguuguaugguu
let-7j-5p	ugagguaguagguuguauaguu
let-7l-5p	ugagguagucgguuguauuguu
miR-1	uggaauguaaagaaguauggag
miR-10	acccuguagauccgaauuugu
miR-100	aacccguaaauccgaacuugug
miR-100-5p	aacccguaaauccgaacuugug
miR-10-3p	caaauucgguucuagagagguuu
miR-10-5p	acccuguagauccgaauuuguu
miR-10a	caaauucgguucuagagagguuu
miR-10a-5p	uacccuguagauccgaauuugug
miR-10d	cacccuguagauccgaauuugu
miR-10d-5p	uacccuguagauccgaauuugu
miR-11	caucacagucugaguucuugc
miR-11-3p	caucacagucugaguucuugc
miR-125	ucccugagacccuaacuuguga
miR-125-5p	ucccugagacccuaacuuguga
miR-125a	ucccugagacccuaacuuguga
miR-125a-5p	ucccugagacccuaacuugcga
miR-125b	ucccugagacccuaacuuguga
miR-125b-5p	ucccugagacccuaacuuguga
miR-125c	ucccugagacccuaacuugugac
miR-1260	aucccaccgcugccacca
miR-13	uaucacagccauuuugacgagu
miR-13-3p	uaucacagccauuuugacgaguu
miR-13a	uaucacagccauuuugacgaguu
miR-13b	uaucacagccauuuugacgagu
miR-13b-3p	uaucacagccauuuugacgagu
miR-1-3p	uggaauguaaagaaguauggag
miR-14	ucagucuuuuucucucucca
miR-14-3p	ucagucuuuuucucucuccau
miR-184	uggacggagaacugauaagggc

miR-184-3p	uggacggagaacugauaagggc
miR-184a	uggacggagaacugauaaggg
miR-184b	uggacggagaacugauaagggc
miR-190	agauauguuugauauucuugguug
miR-190-5p	agauauguuugauauucuugguug
miR-1a	uggaauguaaagaaguauggag
miR-1a-3p	uggaauguaaagaaguauggag
miR-1b	uggaauguaaagaaguaugggu
miR-2	uaucacagccagcuuugaugagc
miR-216a	uaaucucagcugguaauucugag
miR-216a-5p	uaaucucagcugguaauucugag
miR-216b	uaauaucagcugguaauucuga
miR-2-3p	uaucacagccagcuuugaugagc
miR-2478	guaucccacuucugacacca
miR-252	cuaaguacuagugccgcaggag
miR-252-5p	cuaaguacuagugccgcaggag
miR-252a	cuaaguacuagugccgcaggagu
miR-252a-5p	cuaaguacuagugccgcagg
miR-263	aauggcacuggaagaauucacgg
miR-263a	guuaauggcacuggaagaauucac
miR-263a-5p	aauggcacuggaagaauucacggg
miR-263b	cuuggcacugggagaauucac
miR-263b-5p	cuuggcacugggagaauucac
miR-275	ucagguaccugaaguagcgcgcg
miR-275-3p	ucagguaccugaaguagcgcgcg
miR-275-5p	cgcgcuaaucagugaccggggcu
miR-276	uaggaacuucauaccgugcucu
miR-276-3p	uaggaacuucauaccgugcucu
miR-276-5p	agcgagguauagaguuccua
miR-276a	uaggaacuucauaccgugcucu
miR-276a-3p	uaggaacuucauaccgugcucu
miR-276a-5p	cagcgagguauagaguuccuacg
miR-276b	uaggaacuuaauaccgugcucu
miR-276b-3p	uaggaacuuaauaccgugcucu
miR-276b-5p	cagcgagguauagaguuccuacg
miR-277	uaaaugcacuaucugguacgaca
miR-277-3p	uaaaugcacuaucugguacgaca
miR-2779	auccggcucgaaggacca
miR-278	ucggugggacuuucguccguuu
miR-278-3p	ccggaugaugguucacaacgacc
miR-279	ugacuagauccacacucauuaa

**miR-279-3p**	ugacuagauccacacucauuaa
**miR-279-5p**	agugagugagggguccaguguuucaca
**miR-279a**	ugacuagauccacacucaucca
**miR-279a-3p**	ugacuagauccacacucauuaa
**miR-279b-3p**	ugacuagauccacacucaucca
**miR-279d-3p**	ugacuagauccacacucaucca
**miR-281**	ugucauggaauugcucucuuugu
**miR-281-1-5p**	aaagagagcuguccgucgacagu
**miR-281-2-5p**	aagagagcuauccgucgacagu
**miR-281-3p**	ugucauggaauugcucucuuugu
**miR-281-5p**	aagagagcuauccgucgac
**miR-283**	uaaauaucagcugguaauucu
**miR-283-5p**	aaauaucagcugguaauucugg
**miR-2a**	uaucacagccagcuuugaugagc
**miR-2a-2**	ucacagccagcuuugaugagcua
**miR-2a-3p**	uaucacagccagcuuugaugagc
**miR-2b**	uaucacagccagcuuugaggagc
**miR-2b-3p**	uaucacagccagcuuugaggagc
**miR-2c**	uaucacagccagcuuugaugggc
**miR-2c-3p**	uaucacagccagcuuugaugggc
**miR-2d-3p**	uaucacagccagcuuugaugagc
**miR-2g-3p**	uaucacagccagcuuugaugag
**miR-305**	auuguacuucaucaggugcucug
**miR-305-5p**	auuguacuucaucaggugcucug
**miR-31**	uggcaagauguuggcauagcuga
**miR-31-5p**	uggcaagauguuggcauagcua
**miR-316**	uggcaagauguuggcauagcua
**miR-317-5p**	cgggauacacccugugcucgcuuugc
**miR-318**	ucacugggcuuuguuuaucuca
**miR-318-3p**	ucacugggcuuuguuuaucuca
**miR-31a**	uggcaagaugucggcauagcuga
**miR-31a-5p**	uggcaagaugucggcauagcuga
**miR-31b**	uggcaagaugucggaauagcug
**miR-33**	aggugcauuguagucgcauug
**miR-33-5p**	gugcauuguagucgcauuguc
**miR-339**	ucccuguccuccaggagcuca
**miR-34**	uggcagugugguuagcugguug
**miR-34-3p**	cagccacuaucuucacugccgcc
**miR-34-5p**	uggcagugugguuagcugguugug
**miR-34a-5p**	uggcagugugguuagcugguug

miR-34b-5p	uggcaguguaguuagcugguug
miR-34c-5p	uggcagugugauuagcugguug
miR-375	uuuguucguuuggcuuaaguua
miR-375-3p	uuuguucguuuggcuuaaguua
miR-486	uccuguacugagcugccccgag
miR-486-2	uccuguacugagcugccccgagc
miR-486-5p	uccuguacugagcugccccgag
miR-486a	uccuguacugagcugccccgagg
miR-486a-5p	uccuguacugagcugccccgag
miR-486b	uccuguacugagcugccccgagc
miR-486b-5p	uccuguacugagcugccccgag
miR5658	augaugaugaugaugaugaaa
miR-7	uggaagacuagugauuuuguugu
miR-7550	auccggcucgaaggacca
miR-7550-5p	cuccggcucgaaggacca
miR-7-5p	uggaagacuagugauuuuguugu
miR-7a	uggaagacuagugauuuuguugu
miR-7a-5p	uggaagacuagugauuuuguugu
miR-7b	uggaagacuagugauuuuguugu
miR-7b-5p	uggaagacuagugauuuuguuguu
miR-7c	uggaagacuagugauuuuguuguuc
miR-7c-5p	uggaagacuagugauuuuguugu
miR-8	uaauacugucagguaaagaugu
miR-8-3p	uaauacugucagguaaagauguc
miR-8-5p	caucuuaccgggcagcauuaga
miR-9	ucuuugguuaucuagcuguauga
miR-9-1	ucuuugguuaucuagcuguauga
miR-92	auugcacuugucccggccu
miR-9-2	ucuuugguuaucuagcuguauga
miR-927	uuuagaauuccuacgcuuuaccg
miR-927-5p	uuuagaauuccuacgcuuuacc
miR-927a-5p	uuuagaauuccuacgcuuua
miR-92a	uauugcacuugucccggccuau
miR-92a-3p	cgguacggacaggggcaacauu
miR-92b	aauugcacuagucccggccugc
miR-92b-3p	aauugcacuagucccggccugc
miR-9-3p	ucuuugguuaucuagcuguaug
miR-956-3p	uuucgagaccacucuaauccauu
miR-956-5p	guguuuggaauggucucguuagcu
miR-958-3p	ugagauucuucuauucuacuuu
miR-9-5p	ucuuugguauucuagcuguaga

miR-965	uaagcguauagcuuuuccccuu
miR-965-3p	gggguaaaacuguacguuauaug
miR-970	ucauaagacacacgcggcuau
miR-970-3p	ucauaagacacacgcggcuau
miR-988	ccccuuguugcaaaccucacgc
miR-988-3p	ccccuuguugcaaaccucacgc
miR-993	gaagcucgucucuacagguaucu
miR-993-3p	gaagcucgucucuacagguaucu
miR-993a-3p	gaagcucgucucuacagguaucu
miR-993b-3p	gaagcucgucucuacagguaucu
miR-995	ucccugagacccuaacuuguga
miR-995-3p	uagcaccacaugauucggcuu
miR-996	ggcgaacauggaucuagugcacg
miR-996-5p	gcgaacauggaucuagugcacg
miR-998	uagcaccaugagauucagcuc
miR-998-3p	uagcaccaugagauucagcuc
miR-998-5p	acugaauucucgugggucugca
miR-999	uguuaacuguaagacugugucu
miR-9993b-3p	aucucgcuggggccucca
miR-999-3p	uguuaacuguaagacugugucu
miR-9995-3p	aucucgguggaaccucca
miR-9a	ucuuugguuaucuagcuguauga
miR-9a-5p	ucuuugguuaucuagcuguauga
miR-9b	ucuuuggugauuuuagcuguaug
miR-9b-5p	ucuuuggugauuuuagcuguaug
miR-9c	ucuuugguauucuagcuguaga
miR-9c-5p	ucuuugguauucuagcuguaga

S. Table 5

miRNA	Mature miRNA	Fold Difference in Expression Mated Bacteriome to Aposymbiotic tissue
miR-10	acccuguagauccgaauuugu	Positive, 22.03, 7.29
miR-100-5p	aacccguaaauccgaacuugug	Positive 2.76, 4.94
miR-10-3p	caaauucgguucuagagagguuu	Positive, 23.50, 2.98
miR-10a-5p	uacccuguagauccgaauuugug	Positive, 5.64, 5.41
miR-11	caucacagucugaguucuugc	Positive, 2.64, 3.78
miR-13	uaucacagccauuuugacgagu	Positive, 14.74, 2.50
miR-184b	uggacggagaacugauaagggc	Positive, 9.65, 2.19
miR-1b	uggaauguaaagaaguaugggu	Negative, 0.20, 0.28
miR-2	uaucacagccagcuuugaugagc	Positive, 52.48, 29.49
miR-216a	uaaucucagcugguaauucugag	Positive 25.77, 14.13
miR-216b	uaauaucagcugguaauucuga	Positive, 19.44, 6.25
miR-275	ucagguaccugaaguagcgcgcg	Positive, 5.69, 2.39
miR-277	uaaaugcacuaucugguacgaca	Positive, 3.55, 3.76
miR-2779	auccggcucgaaggacca	Positive, 2.48, 2.64
miR-278	ucggugggacuuucguccguuu	Positive, 2.12, 2.31
miR-281	ugucauggaauugcucucuuugu	Positive, 7.37, 6.75
miR-281-1-5p	aaagagagcuguccgucgacagu	Positive, 99.29, 6.73
miR-281-2-5p	aagagagcuauccgucgacagu	Positive, 16.75, 5.03
miR-283	uaaauaucagcugguaauucu	Positive, 28.03, 12.57
miR-283-5p	aaauaucagcugguaauucugg	Positive, 28.03, 12.57
miR-2a	uaucacagccagcuuugaugagc	Positive, 7.44, 29.04
miR-2a-2	ucacagccagcuuugaugagcua	Positive, 51.10, 29.30
miR-305	auuguacuucaucaggugcucug	Positive 92.39, 56.46
miR-316	uggcaagauguuggcauagcua	Positive, 7.27, 8.35
miR-318	ucacugggcuuuguuuaucuca	Positive, 5.35, 10.17
miR-31a	uggcaagaugucggcauagcuga	Positive, 30.03, 104.12
miR-7550	auccggcucgaaggacca	Positive, 2.40, 2.54
miR-8-5p	caucuuaccgggcagcauuaga	Positive, 2.10, 3.22
miR-92	auugcacuugucccggccu	Negative, 0.27, 0.17
miR-92a	uauugcacuugucccggccuau	Negative, 0.27, 0.17
miR-92a-3p	cgguacggacaggggcaacauu	Negative, 0.27, 0.17
miR-956-3p	uuucgagaccacucuaauccauu	Positive, 16.63, 34.04
miR-956-5p	guguuuggaauggucucguuagcu	Positive, 8.24, 6.05
miR-965	uaagcguauagcuuuuccccuu	Positive, 18.78, 19.45
miR-965-3p	gggguaaaacuguacguuauaug	Positive, 19.25, 20.46
miR-993	gaagcucgucucuacagguaucu	Positive, 3.08, 8.25

**miR-995**	**ucccugagacccuaacuuguga**	**Positive, 9.66, 114.17**
**miR-995-3p**	**uagcaccacaugauucggcuu**	**Positive, 9.66, 152.31**
**miR-998-3p**	**uagcaccaugagauucagcuc**	**Positive, 5.00, 2.99**
**miR-9993b-3p**	**aucucgcuggggccucca**	**Negative, 0.31, 0.23**
**miR-9a**	**ucuuugguuaucuagcuguauga**	**Negative, 0.19, 0.07**

S. Table 6

Scaffold location	miRDeep2 score	Estimated probability that the miRNA candidate is a true positive	Total read count	Mature read count	Loop read count	Star read count	Significant randfold p-value	Mature sequence	Consensus star sequence	Consensus precursor sequence
GmorY1:scf7180000652160:1:6287196:1_REF_1359	3.40E+01	0.97 ± 0.03	69	63	0	6	no	aucgaucgcgcugucugggauu	ucccaagcagcgugaucgcg	ucccaagcagcgugaucgcguuguuuaaauuuuauuucaaucgaucgcgcugucugggauu

GbreI1:Scaffold231:1:304319:1_REF_7034	3.10E+02	0.98 ± 0.03	659	614	0	45	no	ugaugaugaugaugaugauga	ugaugaugaugaugaugaugc	ugaugaugaugaugaugaugaugaugaugaugaugaugaugaugaugaugaugaugaugaugaugaugc
GbreI1:Scaffold75:1:1011190:1_REF_4363	1.10E+01	0.98 ± 0.03	25	19	0	6	no	agcgagguauagaguuccuacg	uaggaacuuaauaccgugcucu	agcgagguauagaguuccuacguucgaauauuuuaaauaauucguaggaacuuaauaccgugcucu
GbreI1:Scaffold49:1:1445822:1_REF_3302	4.8	0.98 ± 0.03	12	10	0	2	no	ggcgaacauggaucuagugcacg	ugacuagauuucaugcucgucua	ggcgaacauggaucuagugcacgguuuauucauaaucaaguucgugacuagauuucaugcucgucua

S. Table 7

G. morsitans Gene Id	*G. brevipalpis* Gene ID	miRNA Interactions in *G. morsitans*	miRNA Interactions in *G. brevipalpis*	Interactions Occurring in Both Species
GMOY009401	GBRI041870	miR-316, miR-31a	miR-316, miR-31a	miR-316, miR-31a
GMOY006101	GBRI004801	miR-1b, miR-281, miR-956-5p	miR-956-5p	miR-956-5p
GMOY010240	GBRI003961	miR-316, miR-31a, miR-956-5p	miR-9a	
GMOY000698	GBRI022302		miR-1b, miR-956-5p	
GMOY010090	GBRI030396	miR-184b, miR-92, miR-92a, miR-965-3p	miR-993	
GMOY009161	GBRI028525	miR-10-3p, miR-184b, miR-278, miR-305, miR-956-5p	miR-956-5p, miR-965-3p	miR-956-5p
GMOY005527	GBRI019337	miR-275, miR-92, miR-956-5p	miR-278, miR-281-2-5p, miR-956-5p	miR-956-5p
GMOY006765	GBRI007097	miR-956-5p	miR-184b, miR-2, miR-281-1-5p, miR-92a-3p, miR-956-5p	miR-956-5p
GMOY005029	GBRI012464	miR-965-3p		
GMOY001469	GBRI019785		miR-275	
GMOY004042	GBRI042772	miR-10, miR-316, miR-318, miR-31a, miR-956-5p	miR-316, miR-31a, miR-92a, miR-956-5p	miR-316, miR-31a, miR-956-5p
GMOY003408	GBRI023418	miR-278, miR-281-2-5p, miR-956-3p, miR-956-5p, miR-998-3p, miR-9a	miR-92a	
GMOY000180	GBRI014796	miR-956-5p	miR-281	
GMOY012057	GBRI042754		miR-184b, miR-281-1-5p, miR-281-2-5p, miR-305	
GMOY001137	GBRI044528	miR-184b, miR-2, miR-275, miR-316, miR-31a	miR-184b, miR-275, miR-316, miR-31a, miR-92a-3p, miR-956-5p, miR-9a	miR-184b, miR-275, miR-316, miR-31a
GMOY008996	GBRI040391	miR-281-1-5p	miR-956-5p	
GMOY007451	GBRI043918	miR-11, miR-278, miR-316, miR-31a, miR-956-5p	miR-11	miR-11
GMOY010676	GBRI002237	miR-1b	miR-316, miR-31a	
GMOY000069	GBRI041971	miR-318, miR-956-5p	miR-100-5p, miR-9a	

S. Fig. 1

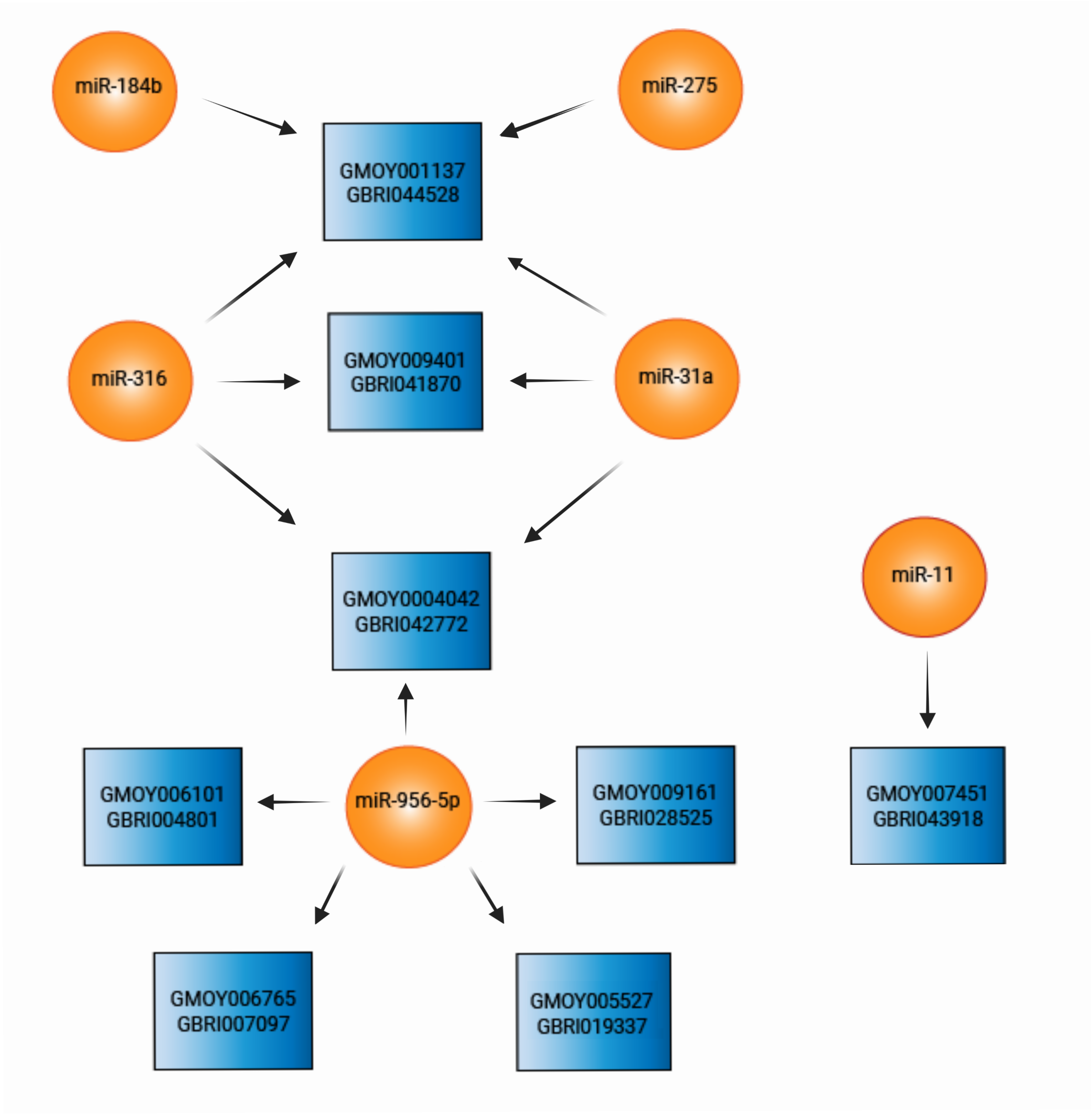

miR-184b
miR-275
GMOY001137
GBRI044528
miR-316
GMOY009401
GBRI041870
miR-31a
GMOY0004042
GBRI042772
miR-11
GMOY006101
GBRI004801
miR-956-5p
GMOY009161
GBRI028525
GMOY007451
GBRI043918
GMOY006765
GBRI007097
GMOY005527
GBRI019337

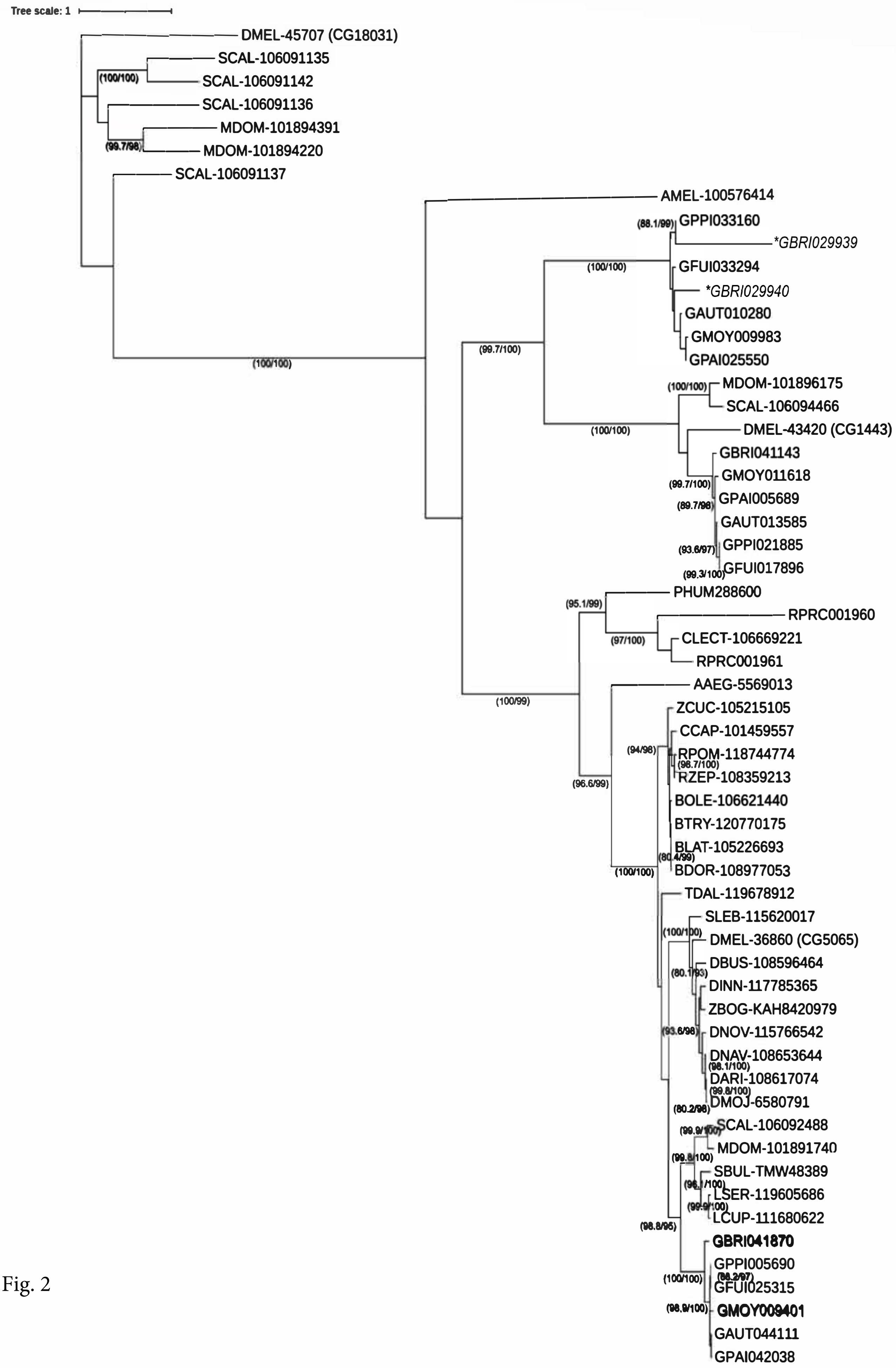
Tree scale: 1
DMEL-45707 (CG18031)
SCAL-106091135
SCAL-106091142
SCAL-106091136
MDOM-101894391
MDOM-101894220
SCAL-106091137
AMEL-100576414
GPPI033160
*GBRI029939
GFUI033294
*GBRI029940
GAUT010280
GMOY009983
GPAI025550
MDOM-101896175
SCAL-106094466
DMEL-43420 (CG1443)
GBRI041143
GMOY011618
GPAI005689
GAUT013585
GPPI021885
GFUI017896
PHUM288600
RPRC001960
CLECT-106669221
RPRC001961
AAEG-5569013
ZCUC-105215105
CCAP-101459557
RPOM-118744774
RZEP-108359213
BOLE-106621440
BTRY-120770175
BLAT-105226693
BDOR-108977053
TDAL-119678912
SLEB-115620017
DMEL-36860 (CG5065)
DBUS-108596464
DINN-117785365
ZBOG-KAH8420979
DNOV-115766542
DNAV-108653644
DARI-108617074
DMOJ-6580791
SCAL-106092488
MDOM-101891740
SBUL-TMW48389
LSER-119605686
LCUP-111680622
GBRI041870
GPPI005690
GFUI025315
GMOY009401
GAUT044111
GPAI042038
(100/100)
(99.7/98)
(100/100)
(88.1/99)
(100/100)
(99.7/100)
(100/100)
(100/100)
(99.7/100)
(89.7/98)
(93.6/97)
(99.3/100)
(95.1/99)
(97/100)
(100/99)
(96.6/99)
(94/98)
(96.7/100)
(100/100)
(100/100)
(98.8/95)
(100/100)
(98.9/100)

S. Fig. 2
